# FTO regulates ELK3-mediated metabolic rewiring and represents a unique therapeutic target in T cell leukemia

**DOI:** 10.1126/sciadv.adq3052

**Published:** 2025-05-28

**Authors:** Hao Huang, Xinlu Li, Jinlian Luo, Chuan Gao, Mengjie Yang, Jin Xu, Ting Xie, Zhi Chen, Donghai Wang, Yuan Wang, Hua-Bing Li, Jinyan Huang, Yu Liu, Haojian Zhang, Panagiotis Ntziachristos, Yun Zhao, Guoliang Qing, Hudan Liu

**Affiliations:** ^1^ Department of Hematology, Zhongnan Hospital of Wuhan University, State Key Laboratory of Metabolism and Regulation in Complex Organisms, Wuhan University, Wuhan, China.; ^2^ Frontier Science Center of Immunology and Metabolism, Medical Research Institute, Wuhan University, Wuhan, China.; ^3^ TaiKang Centre for Life and Medical Sciences, Wuhan University, Wuhan, China.; ^4^ Department of Physiology, School of Basic Medical Sciences, Wuhan University, Wuhan, China.; ^5^ Shanghai Institute of Immunology, State Key Laboratory of Oncogenes and Related Genes, Shanghai Jiao Tong University School of Medicine, Shanghai, China.; ^6^ Center for Biomedical Big Data, The First Affiliated Hospital, School of Medicine, Zhejiang University, Hangzhou, China.; ^7^ Pediatric Translational Medicine Institute, Shanghai Children’s Medical Center, School of Medicine, Shanghai Jiao Tong University, Shanghai, China.; ^8^ Leukemia Therapy Resistance Lab, Department of Biomolecular Medicine, Faculty of Medicine and Health Sciences, Ghent University, Ghent, Belgium.; ^9^ Cyrus Tang Medical Institute, National Clinical Research Center for Hematologic Diseases, Soochow University, Suzhou, China.

## Abstract

Understanding the regulation of N6-methyladenosine (m^6^A), the prominent internal modification in mRNA, fosters the development of potential therapeutic strategies for human cancers. While the m^6^A demethylases FTO and ALKBH5 are recognized for their crucial roles in various cancers, their impact on lymphoid leukemia remains uncertain. Using T cell acute lymphoblastic leukemia (T-ALL) as a model system, we identify FTO as a unique vulnerability in T cell leukemia. Knockout of *FTO*, but not *ALKBH5*, significantly suppresses leukemia initiation and progression. Mechanistic analysis reveals that FTO heightens *ELK3* mRNA stability in an m^6^A-dependent manner. Elevated ELK3 in turn transcriptionally activates the expression of glycolytic genes. Pharmacological inhibition of FTO suppresses *ELK3* expression, hampers glycolysis and manifests remarkable antileukemia efficacy. Our findings unravel the crucial role of FTO in T-ALL and highlight the FTO-ELK3 axis as a key nodule during leukemogenesis, thereby providing a fundamental basis to harness selective FTO antagonist for T-ALL therapeutics.

## Teaser

The RNA m^6^A demethylase FTO represents a promising therapeutic target in T cell leukemia.

## INTRODUCTION

T cell acute lymphoblastic leukemia (T-ALL) is a lethal and aggressive hematological malignancy characterized by aberrant proliferation of immature thymocytes that afflicts both children and adults (*1*). Although overall survival (OS) of 80% in the pediatric setting has been achieved using intensive multi-agent combination chemotherapeutic protocols, it is often accompanied by substantial short-term and long-term side effects (*2*, *3*). Moreover, OS rates for adult patients with T-ALL are lower than 50% due to higher treatment-related toxicities (*4*). A considerable proportion of patients with T-ALL confront the challenge of disease relapse, with a particularly grim prognosis for cases resistant to chemotherapy (*5*). These clinical challenges underscore an urgent need for the implementation of targeted therapies. Molecular genetic analyses and sequencing studies have identified genetic abnormality involved in T-ALL development (*6*). However, clinically approved targeted treatment options for patients with T-ALL remain limited, highlighting the requirement for a more in-depth understanding of the molecular pathogenesis that may yield potential effective therapeutic strategies.

N^6^-methyladenosine (m^6^A) is the most prominent internal modification in mRNA that regulates gene expression at the post-transcriptional level (*7*, *8*). The m^6^A modification is deposited on mRNA by the methyltransferase-like 3 (METTL3)-methyltransferase-like 14 (METTL14)-Wilms’s tumor 1-associating protein (WTAP) methyltransferase complex and can be reversibly removed by demethylases alkB homolog 5 (ALKBH5) or fat mass- and obesity-associated protein (FTO) (*9*). This modification plays a crucial role in determining the fate of modified RNA molecules, influencing various aspects of RNA metabolism. In many cases, it governs mRNA stability and translation, contributing to the regulatory landscape of cellular processes (*7*, *8*). As such, the RNA m^6^A modification is considered as a prominent epigenetic regulation of gene expression, implicated in many pathophysiological processes including cancer (*10*). Accumulating experimental data have demonstrated that the global abundance of m^6^A, as well as the expression levels of methylases and demethylases, is dysregulated in human cancer. These factors are crucial for cancer initiation and progression (*11*–*15*). Yet, the functional significance of these m^6^A regulators in T-ALL remains unclear.

In this study, we systematically analyzed the genes encoding m^6^A writer and eraser proteins in primary T-ALL samples and revealed that the m^6^A demethylases, FTO and ALKBH5, are more abundant in transformed thymocytes. These results suggest that RNA m^6^A demethylation plays a vital role in T cell leukemogenesis. To provide proof of concept, we used the T cell–specific lineage knockout mouse strains that delete *Fto* or *Alkbh5* to establish NOTCH1-driven T-ALL. T cell leukemogenesis is strictly dependent on FTO, as ablation of *FTO* but not *ALKBH5* significantly blocks T-ALL onset and progression. Our multi-omics integration revealed *ELK3* as a pivotal FTO downstream target. FTO-mediated removal of m^6^A from ETS domain-containing protein Elk-3 (ELK3) mRNA enhances its stability, leading to increased levels of ELK3, which in turn activates the transcription of glycolysis-related genes, such as phosphoglycerate kinase 1 (PGK1), thereby promoting glycolysis in T-ALL. With several FTO inhibitors advancing to clinical stages, our findings suggest that these small-molecule drugs could be exploited for the treatment of T cell leukemia.

## RESULTS

### *Fto* is selectively required for murine T-ALL development

**Fig. 1.**
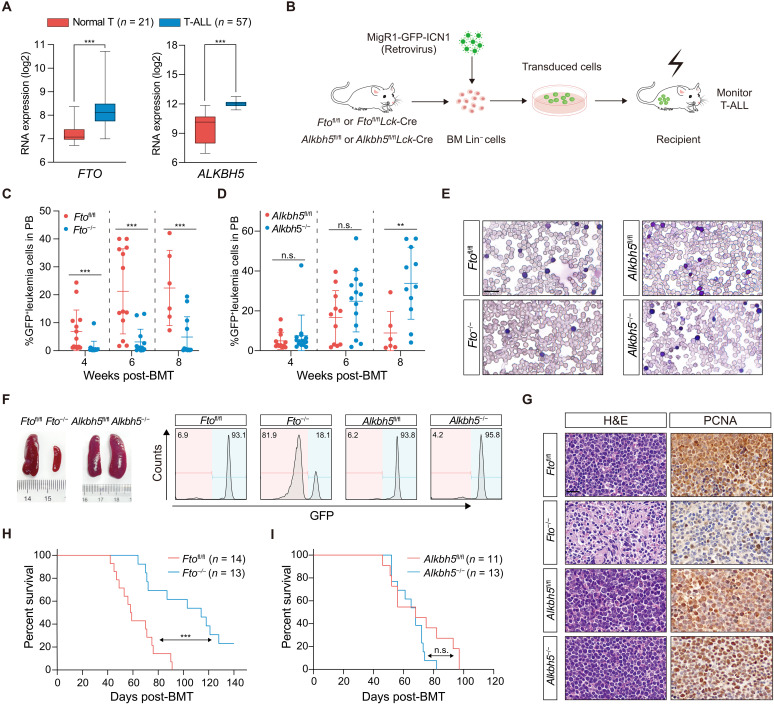
Fto is required for NOTCH1-induced murine T-ALL development. (**A**) Expression analysis of *FTO* and *ALKBH5* among 57 primary patients with T-ALL and 21 normal T cells from GSE33469 and GSE33470. The expression values are presented as box-and-whisker plots with a log2 scale. (**B**) Experimental scheme for intracellular NOTCH1 (ICN1)–induced T-ALL model. Bone marrow (BM) lineage-negative (Lin^−^) cells were isolated from the mice as depicted and infected with MigR1-GFP-ICN1 retrovirus and then transplanted into irradiated recipient mice. (**C** and **D**) Percentage of GFP^+^ leukemia cells in peripheral blood (PB) of recipient mice at the indicated time after BM transplantation (BMT). Recipient mice were transplanted with ICN1-transduced *Fto*^fl/fl^ (*n* = 14), *Fto*^−/−^ (*n* = 16) (C), *Alkbh5*^fl/fl^ (*n* = 12), or *Alkbh5*^−/−^ (*n* = 14) (D) BM Lin^−^ cells as indicated. (**E**) Representative images of Wright-Giemsa staining of PB from recipient mice as indicated. Mice were euthanized at the same time when the *Fto*^fl/fl^ or *Alkbh5*^fl/fl^ control became moribund. The scale bar represents 20 μm. (**F**) Spleen image (left) and flow cytometry analysis of GFP^+^ leukemia cells in the spleen (right) are shown. (**G**) Hematoxylin and eosin (H&E) staining (left) and PCNA immunohistological images (right) in spleen sections from indicated mice. The scale bar represents 20 μm. PCNA, proliferating cell nuclear antigen. (**H** and **I**) Kaplan-Meier survival curves for recipient mice transplanted with ICN1-transduced BM Lin^−^ cells from *Fto*^fl/fl^ (*n* = 14), *Fto^−/−^* (*n* = 13) (H), *Alkbh5*^fl/fl^ (*n* = 11), or *Alkbh5*^−/−^ (*n* = 13) (I). Data are represented as means (±SD). Data were analyzed by Welch *t* test (A), Mann-Whitney test [(C) and (D)], and log-rank test [(H) and (I)]. **P < 0.01 and ***P < 0.001. n.s., nonsignificant.

We compared the expression of m^6^A modulators in 57 primary T-ALL specimens with 21 normal thymocyte samples (*16*) and observed that both m^6^A demethylases (FTO and ALKBH5) were significantly up-regulated in T-ALL (Fig. 1A). Expression changes of the methyltransferase complex components (METTL3 and WTAP) were moderate and insignificant, and METTL14 was even decreased in transformed T cells (fig. S1A), suggesting that RNA m^6^A modifications become less prevalent during transformation. In line with these data, m^6^A dot blots confirmed weaker m^6^A signals in murine T-ALL cells as compared with normal thymocytes (fig. S1B). Analysis of gene expression profiles from additional primary T-ALL samples revealed that elevated *FTO* and *ALKBH5* expression is not restricted to any specific T-ALL subtype nor is it associated with common mutations found in T-ALL (fig. S1, C and D) (*17*–*24*).

We hypothesized that FTO and ALKBH5 play crucial proleukemogenic roles in T-ALL, akin to their functions in acute myeloid leukemia (AML) (*12*–*14*). To test the notion, we obtained *Fto*^fl/fl^ and *Alkbh5*^fl/fl^ mice and bred them with the *Lck*-Cre strain to generate *Fto*^fl/fl^*Lck*-Cre or *Alkbh*^fl/fl^*Lck*-Cre (hereafter referred to as “*Fto*^−/−^” and “*Alkbh5*^−/−^”) mouse strains that allow specific deletion of *Fto* or *Alkbh5* in T cell lineage (fig. S2, A to D). To establish NOTCH1-induced T-ALL murine model (*25*), we transduced MigR1–ICN1 (intracellular NOTCH1) retroviruses, with green fluorescent protein (GFP) as a surrogate marker, into bone marrow (BM) lineage negative (Lin^−^) cells from *Fto*^fl/fl^, *Fto^−/−^*, *Alkbh5*^fl/fl^, or *Alkbh5*^−/−^ mice and transplanted these cells into irradiated recipient mice (Fig. 1B). In comparison to the *Fto*^fl/fl^ control cohort, deletion of *Fto* resulted in a significantly lower percentage of GFP^+^ leukemia cells, which were also CD4^+^CD8^+^ T cell blasts, in the peripheral blood (PB) after BM transplantation (BMT) (Fig. 1C and fig. S2E). Unexpectedly, the deficiency of *Alkbh5* did not suppress the peripheral production of GFP^+^ leukemia cells (Fig. 1D). Wright-Giemsa staining of PB smears corroborated this finding (Fig. 1E). Deletion of *Fto* resulted in less severe splenomegaly and fewer disseminated leukemia cells in the spleen. Conversely, *Alkbh5* knockout did not decrease the spleen size nor the infiltration of leukemia cells in the spleen (Fig. 1F and fig. S2F). Hematoxylin and eosin (H&E) and immunohistochemical staining of proliferating cell nuclear antigen (PCNA) further demonstrated that deficiency of *Fto* instead of *Alkbh5* restrained leukemia cells infiltration and proliferation (Fig. 1G). As a result, only deletion of *Fto* significantly delayed leukemia onset and prolonged survival in the recipient mice (Fig. 1, H and I). Deficiency of *Alkbh5* or *Fto* in leukemia cells was confirmed by quantitative polymerase chain reaction (qPCR) and immunoblotting (fig. S2G), ruling out the possibility that the phenotypic consequence from *Alkbh5^−/−^* mice was due to incomplete gene knockout. Of note, *Fto* deficiency in T cell lineage did not appreciably affect thymus size, overall thymocyte numbers, or the distribution of T cell progenitor subsets (fig. S3), suggesting an insignificant role of FTO in normal T cell development. Our findings therefore support that FTO presents a unique and specific dependency in T-ALL.

### FTO demethylase activity is required for T-ALL progression

**Fig. 2.**
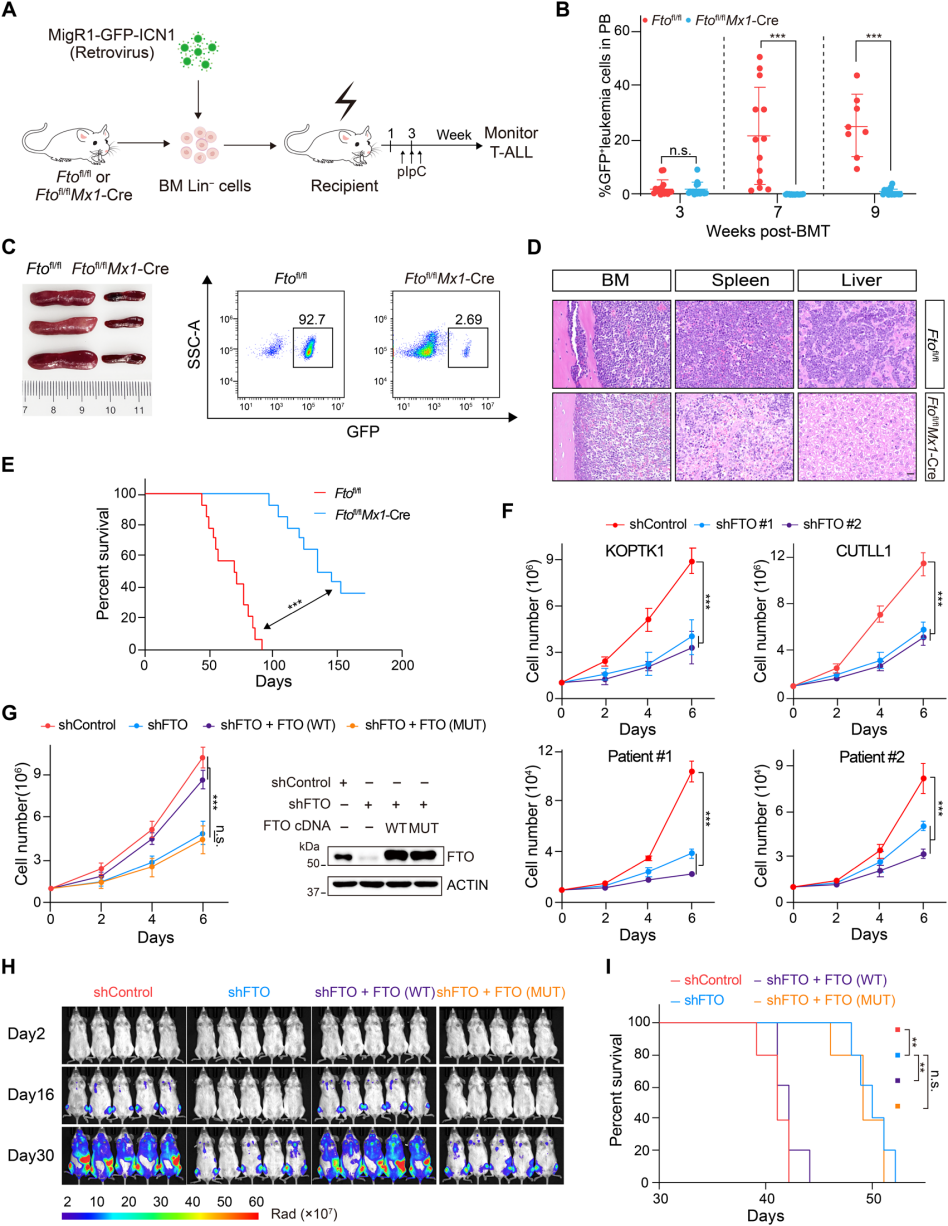
FTO promotes T-ALL in an m^6^A-dependent manner. (**A**) Experimental scheme for ICN1-induced T-ALL model using *Fto*^fl/fl^ or *Fto*^fl/fl^*Mx1*-Cre mice as donors. BM Lin^−^ cells were isolated and infected with MigR1-GFP-ICN1 retrovirus, followed by transplantation into irradiated recipient mice. pIpC (polyinosinic-polycytidylic acid) (10 mg/kg) was injected 3 weeks later. (**B**) Percentages of GFP^+^ cells in PB of recipient mice at the indicated time after transplantation. *Fto*^fl/fl^ (*n* = 15), *Fto*^fl/fl^*Mx1*-Cre (*n* = 17). (**C**) Spleen image (left) and flow cytometry analysis of GFP^+^ leukemia cells in the spleen (right) of recipients 8 weeks postengraftment. (**D**) Representative images of H&E staining for BM, spleen, and liver of recipients 8 weeks postengraftment. The scale bar represents 20 μm. (**E**) Kaplan-Meier curves of recipient mice transplanted with ICN1-transduced BM Lin^−^ cells from *Fto*^fl/fl^ and *Fto*^fl/fl^*Mx1*-Cre mice (*n* = 14 per group). (**F**) The growth curves of KOPTK1, CUTLL1 cells, and two primary T-ALL cells, following *FTO* knockdown by two independent shRNAs. (**G**) KOPTK1 cell growth upon FTO depletion, with or without the restoration of wild-type (WT) or m^6^A demethylase-inactive mutant (MUT) FTO (left). Protein expression was analyzed by immunoblotting and shown on the right. (**H**) In vivo bioluminescence imaging showing leukemia burden in luciferase expressing KOPTK1 xenograft (*n* = 5 per group). The unit of radiance is “photons/second/cm^2^/steradian”. (**I**) Kaplan-Meier survival curves of KOPTK1 xenografts shown in (H) (*n* = 5 per group). Data are presented as means (±SD) from two biological replicates, each performed with three technical replicates (F and G). Data were analyzed by Mann-Whitney test (B), two-way ANOVA with Bonferroni’s multiple comparisons [(F) and (G)], and log-rank test [(E) and (I)]. **P < 0.01 and ***P < 0.001. n.s., nonsignificant.

We next assessed the role of FTO in T-ALL progression after leukemia onset. *Fto*^fl/fl^ mice were crossed with the *Mx1*-Cre strain (fig. S4A), and the resulting mice were used as donors for MigR1-GFP-ICN1 retroviral transduction and subsequent transplantation. Following the procedure, polyinosinic-polycytidylic acid (pIpC) was intravenously injected to induce *Fto* knockout (Fig. 2A). Consistently, ablation of *Fto* hampered the peripheral production of GFP^+^ leukemia cells and reduced leukemia burden in BM, spleen, and liver (Fig. 2, B to D, and fig. S4B). As a result, *Fto* depletion after leukemia initiation considerably prolonged leukemia mice survival and life span (Fig. 2E). In contrast, conditional *Alkbh5* deletion by *Mx1*-Cre did not affect leukemia cell infiltration in vivo, as well as the OS (fig. S4, C to F).

We then assessed the functional importance of FTO in human T-ALL cells using shRNA-mediated gene silencing. As shown in Fig. 2F and fig. S4G, *FTO* depletion substantially suppressed the growth of human T-ALL KOPTK1 and CUTLL1 cells, as well as primary T-ALL cells from patients (table S1). Cell growth inhibition was associated with a notable increase in global m^6^A levels (fig. S4H), leading us to hypothesize that the FTO enzymatic activity contributes to its proleukemic function. To test this, wild-type (WT) FTO or the enzymatically inactive mutant (MUT) FTO (H231A/D233A) (*12*) was expressed in *FTO*-depleted cells. Only WT FTO expression was sufficient to rescue the growth defect (Fig. 2G and fig. S4I), reinforcing the essential role of FTO demethylase activity in T-ALL cells.

We further established a human xenograft using KOPTK1 cells expressing firefly luciferase. Equal amounts of *FTO*-depleted cells with or without the exogenous expression of WT/MUT FTO were intravenously injected into immunodeficient NOD.Cg-Prkdcscid Il2rgtm1Vst/Vst (NPG) mice (fig. S4, J and K). *FTO* knockdown markedly inhibited leukemia progression in vivo, as evidenced by much weaker luciferase signals over time compared with the control (Fig. 2H and fig. S2L). As a result, mice transplanted with *FTO*-deficient cells exhibited a significantly extended lifespan (Fig. 2I). Supplementation of WT, but not catalytically inactive FTO, significantly boosted leukemia cell expansion in vivo (Fig. 2H and fig. S4L), leading to shortened life span of leukemia mice (Fig. 2I). These findings consolidate the indispensable role of FTO demethylase activity in T-ALL progression.

### Integrative analysis of differential RNA expression and m^6^A modification identifies potential FTO targets in T-ALL

**Fig. 3.**
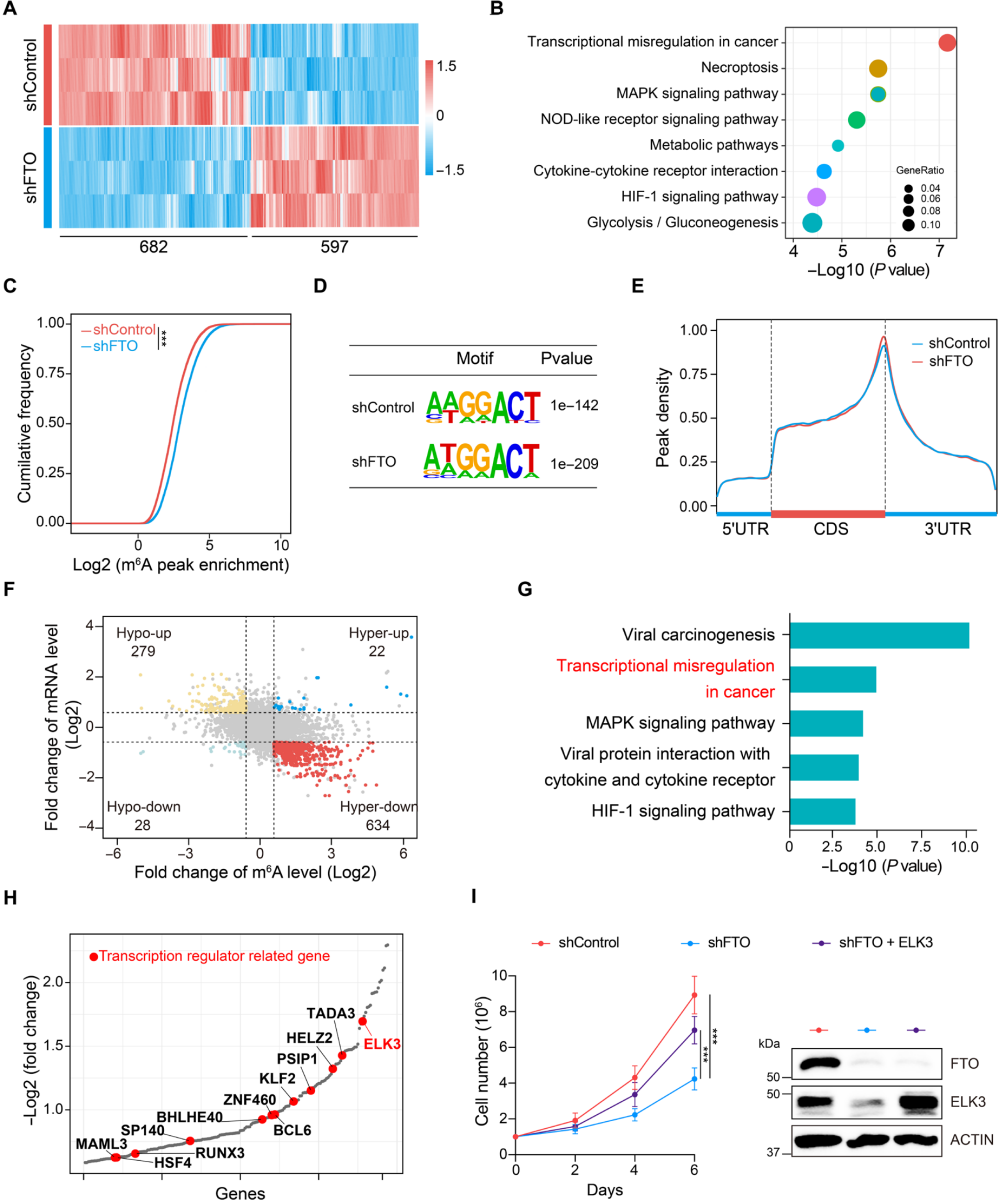
Identification of potential key targets of FTO in T-ALL cells. (**A**) Heatmap showing differentially expressed genes in KOPTK1 cells with or without *FTO* shRNA expression. Differential genes are derived from RNA-seq data and defined as |fold change| ≥ 1.5, *P* value <0.05. (**B**) KEGG enrichment analysis of down-regulated transcripts in *FTO*-depleted KOPTK1 cells. (**C**) Cumulative distribution function representing the log2 peak intensity of m^6^A-modified sites in KOPTK1 cells with or without *FTO* knockdown. The *P* value was calculated using two-tailed Kolmogorov-Smirnov test. (**D**) Top consensus sequence motif identified within m^6^A peaks by hypergeometric optimization of motif enrichment (HOMER) in KOPTK1 cells with or without *FTO* knockdown. (**E**) Metagene profiles of m^6^A peak distribution along a normalized transcript composed of three rescaled nonoverlapping segments: 5′UTR, CDS, and 3′UTR in KOPTK1 cells with or without *FTO* knockdown. (**F**) Distribution of genes exhibiting a significant change in both m^6^A level and overall transcript level in *FTO*-depleted KOPTK1 cells. (**G**) KEGG pathway enrichment analysis with the 634 hyperdown genes shown in (F). (**H**) Bubble-rank plot of hyperdown transcripts in (F). The genes encoding transcription factors are highlighted in red. (**I**) Growth curves of *FTO*-depleted KOPTK1 cells with or without the ectopic expression of *ELK3* (left). Immunoblots of FTO and ELK3 are shown on the right. Data are presented as means (±SD) from two biological replicates, each performed with three technical replicates. Data were analyzed by two-way ANOVA with Bonferroni’s multiple comparisons. ***P < 0.001.

To comprehensively view FTO-regulated RNA transcripts in T-ALL, we performed RNA sequencing (RNA-seq) in KOPTK1 cells with or without *FTO* shRNA expression. A total of 597 genes were significantly up-regulated whereas 682 genes were down-regulated (Fig. 3A and fig. S5A). Differentially expressed genes were subjected to Kyoto Encyclopedia of Genes and Genomes (KEGG) enrichment analysis, and we identified the top eight pathways in which the down-regulated genes were enriched. Notably, *FTO* knockdown caused significant suppression of genes involved in “transcriptional misregulation in cancer”, which ranked at the top (Fig. 3B). We next explored the m^6^A methylomes using methylated RNA immunoprecipitation sequencing (MeRIP-seq/m^6^A-seq) in KOPTK1 cells. Consistent with prior studies in other tumor cells (*26*), *FTO* loss led to a noticeable increase in m^6^A abundance (Fig. 3C). These m^6^A modifications were mapped primarily to the classical DRACH motif (Fig. 3D) and enriched in coding sequences (CDSs), 3′ untranslated regions (3′UTRs) with the abundance reaching peak around stop codons (*27*, *28*) (Fig. 3E and fig. S5B). *FTO* depletion induced 1619 significantly increased peaks (m^6^A-hyper) and 907 decreased signals (m^6^A-hypo) (fig. S5C), highlighting a prominent gain of m^6^A methylation on mRNA transcripts following *FTO* knockdown.

To pinpoint crucial FTO target RNA transcripts whose altered gene expression results from FTO-mediated m^6^A demethylation, we merged differentially expressed genes with those with m^6^A abundance change. Combined analysis of RNA-seq and m^6^A-seq data from KOPTK1 cells identified 656 hypermethylated m^6^A peaks. Among these, the corresponding mRNA transcripts were predominantly down-regulated (634/97%, Hyper-down), while a small fraction were up-regulated (22/3%, Hyper-up) (Fig. 3F). Given their potential role as direct FTO targets with proleukemogenic functions, we focused on the Hyper-down group. KEGG analysis enriched the pathway associated with “transcriptional misregulation in cancer” (Fig. 3G), analogous to the enrichment of FTO-induced genes shown in Fig. 3B. We further conducted a detailed analysis of genes encoding transcription factors, identifying 11 transcription factors in the Hyper-down group (Fig. 3H). Among those, BCL6 has been previously characterized as a proleukemogenic transcription factor in T-ALL (*29*). These results support the effectiveness of our integrative analysis in revealing key T-ALL associated transcription factors. Down-regulation of these genes following *FTO* knockdown were validated by qPCR (fig. S5D).

Notably, ELK3 is the most down-regulated transcription factor among all identified. ELK3, also known as NET, encodes a member of the ETS-domain transcription factor family and the ternary complex factor subfamily (*30*). ELK3 has been identified as a susceptibility locus for childhood acute lymphoblastic leukemia (ALL) (*31*) and several studies showed that *ELK3* plays a critical oncogenic role in various cancers, including glioma, pancreatic, and gastric cancers (*30*). We found *ELK3* expression was significantly elevated in human T-ALL samples (fig. S5E). Overexpression of *ELK3* significantly rescued the growth defect of T-ALL cells caused by *FTO* loss (Fig. 3I and fig. S5F), suggesting that ELK3 is a functionally important downstream target gene regulated by FTO. Consequently, we focused on *ELK3* for further regulatory and functional studies.

### FTO regulates *ELK3* mRNA stability in an m^6^A-dependent manner

**Fig. 4.**
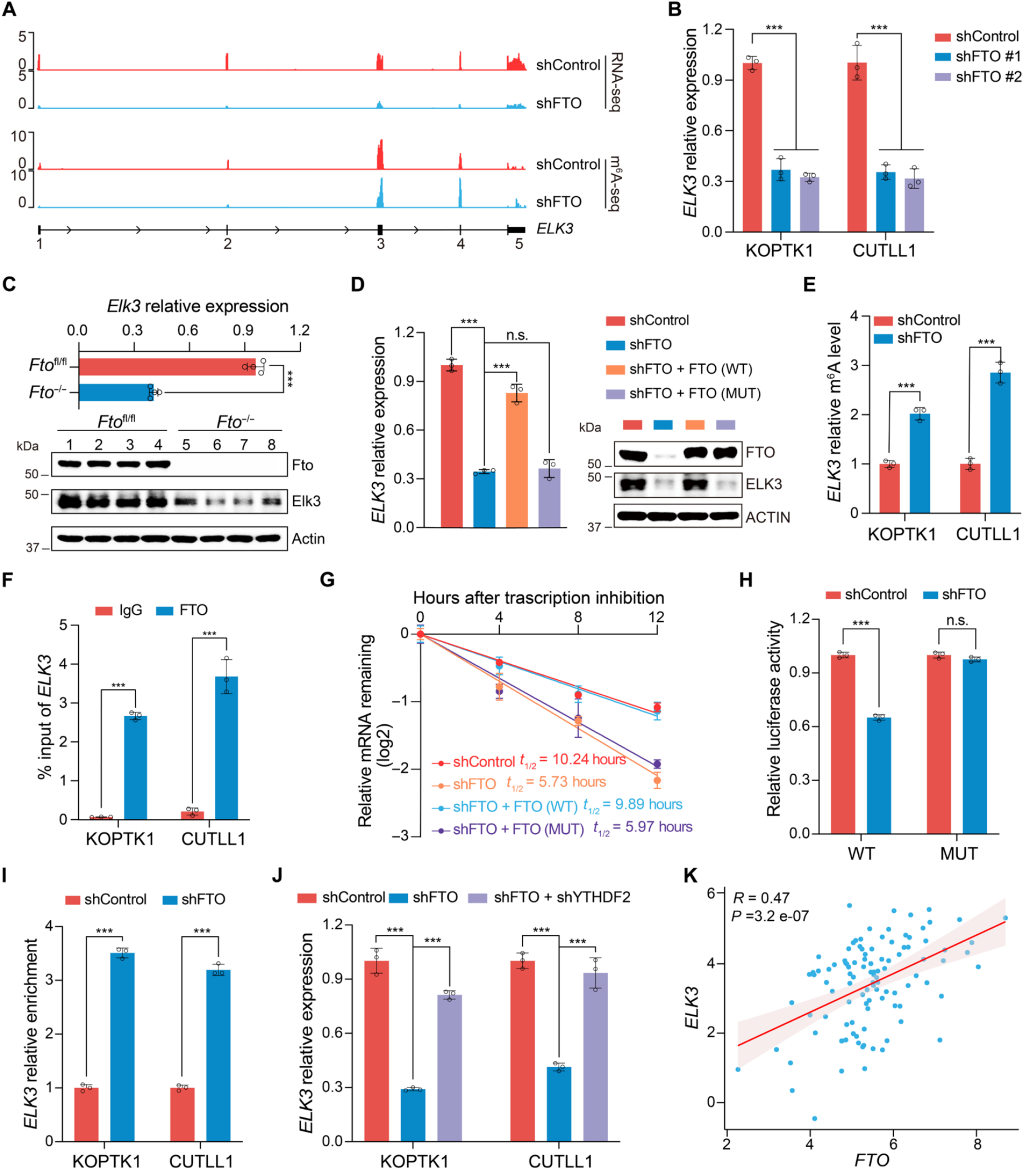
FTO regulates *ELK3* expression through enhancing its mRNA stability. (**A**) Genome browser tracks showing the mRNA abundance and m^6^A peaks of *ELK3* transcripts in KOPTK1 cells with or without *FTO* depletion. (**B**) qPCR analysis of *ELK3* expression in *FTO* knockdown T-ALL cells as indicated. (**C**) mRNA (top) and protein (bottom) levels of *Elk3* in primary murine T-ALL cells from *Fto*^fl/fl^ or *Fto*^−/−^ BMT as shown in fig. S2G (*n* = 4 per group). (**D**) qPCR (left) and immunoblot (right) analysis of *ELK3* expression in *FTO*-depleted KOPTK1 cells rescued with WT or MUT FTO. (**E**) Gene-specific m^6^A-qPCR analysis of the *ELK3* mRNA in T-ALL cells as indicated. (**F**) FTO-RIP qPCR validation of FTO interaction with the *ELK3* mRNA in T-ALL cells. (**G**) The mRNA half-life (*t*_1/2_) of *ELK3* in *FTO* knockdown KOPTK1 cells with enforced expression of WT or MUT FTO. (**H**) Relative luciferase activity showing the WT or MUT *ELK3* exon4 firefly luciferase reporter in 293T cells transduced with Control or FTO shRNA. (**I**) YTHDF2-RIP qPCR showing the binding of YTHDF2 to *ELK3* mRNA in *FTO* knockdown leukemia cells. (**J**) The *ELK3* mRNA level in *FTO*-deficient T-ALL cells with or without *YTHDF2* shRNA expression. (**K**) Scatterplot showing the correlation of *FTO* and *ELK3* in 108 T-ALL samples from databases of National Omics Data Encyclopedia (NODE) (OEP002748). The expression levels were log_2_(TPM). Pearson correlation coefficient (*R*) and *P* value (*P*) are marked. Data are presented as means (±SD) of technical triplicates, and the experiments were independently repeated at least twice with the similar results [(B) and (D) to (J)]. Data were analyzed by one-way ANOVA with Tukey’s multiple comparisons [(B), (D), and (J)], two-way ANOVA with Bonferroni’s multiple comparisons (H), and unpaired two-tailed Student’s *t* test [(C), (E), (F), and (I)]. ***P < 0.001, n.s., nonsignificant.

We examined genome browser tracks and affirmed that depletion of *FTO* significantly attenuated the expression of *ELK3*, concomitant with a notable elevation of the m^6^A peak in the exon 4 (Fig. 4A). The decrease in *ELK3* RNA and protein expression was confirmed in human T-ALL KOPTK1 and CUTLL1 cells following *FTO* knockdown (Fig. 4B and fig. S6A). Consistently, down-regulation of *Elk3* was also found in primary murine *Fto*^−/−^ leukemia cells (Fig. 4C), suggesting that the activation of *ELK3* expression by *FTO* is an evolutionarily conserved regulatory mechanism in human and mouse. Enforced expression of WT *FTO*, but not catalytically inactive mutant, substantially restored *ELK3* expression in *FTO*-depleted cells (Fig. 4D and fig. S6B). We confirmed an increase in *ELK3* m^6^A modifications upon *FTO* knockdown (Fig. 4E), and a direct binding of the *ELK3* transcript by FTO in human T-ALL KOPTK1 and CUTLL1 cells (Fig. 4F). These data suggest that the *FTO*-mediated *ELK3* RNA m^6^A demethylation is responsible for high *ELK3* expression.

Given the strong link between m^6^A modification and mRNA stability (*32*), we treated *FTO*-depleted KOPTK1 and CUTLL1 cells with the transcription inhibitor actinomycin D and measured the half-life of *ELK3* transcripts. *FTO* ablation caused a noticeable decrease in the half-life of *ELK3* mRNA (fig. S6C). Exogenous expression of WT FTO, but not inactive mutant, effectively restored *ELK3* mRNA stability (Fig. 4G and fig. S6D). We also conducted a luciferase reporter assay and observed that *FTO* knockdown significantly decreased the activity of the luciferase construct fused with the *ELK3* exon 4 in-frame but not the mutant sequence with m^6^A site being disrupted (Fig. 4H and fig. S6E). YTH N6-Methyladenosine RNA Binding Protein F2 (YTHDF2) is the primary m^6^A reader that promotes the degradation of the m^6^A-containing RNAs (*33*, *34*). RIP-qPCR analysis confirmed that YTHDF2 interacts with the *ELK3* mRNA (fig. S6F). This RNA binding of YTHDF2 was more profound upon *FTO* knockdown (Fig. 4I). In addition, *YTHDF2* knockdown diminished the effects of *FTO* deficiency on *ELK3* mRNA and protein down-regulation (Fig. 4J and fig. S6G), demonstrating that YTHDF2 is the m^6^A reader for the *ELK3* mRNA, responsible for its m^6^A-modified RNA degradation. We analyzed primary patients with T-ALL samples, and observed that *ELK3* expression was significantly and positively correlated with *FTO* (*17*) (Fig. 4K), further supporting the regulatory role of *FTO* in sustaining *ELK3* expression. In contrast, *ELK3* expression was neither associated with *ALKBH5* in primary human T-ALL (fig. S6H) nor regulated by *Alkbh5* in NOTCH1-driven murine T-ALL cells (fig. S6I). On the basis of these results, we demonstrate that FTO specifically catalyzes the demethylation of *ELK3* mRNA, resulting in its detachment from YTHDF2 and thereby stabilization of the RNA transcript.

### *ELK3* serves as a master regulator promoting glycolysis in T-ALL

**Fig. 5.**
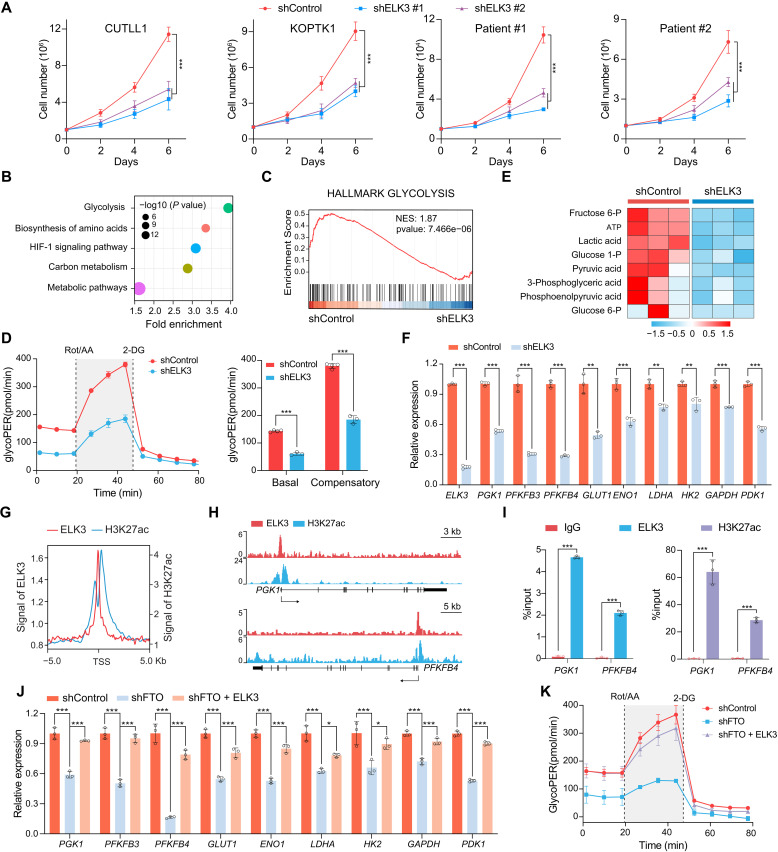
ELK3 acts as a functionally important target of FTO through regulating glycolysis. (**A**) Growth curves of *ELK3*-depleted T-ALL cell lines (CUTLL1 and KOPTK1) and primary T-ALL cells. Data are presented as the mean ± SD from two biological replicates, each with three technical replicates. (**B**) KEGG pathway analysis of down-regulated genes in *ELK3*-depleted KOPTK1 cells. (**C**) GSEA plot showing enrichment of gene sets of hallmark glycolysis in *ELK3* knockdown CUTLL1 cells. (**D**) Glycolytic proton efflux rate (glycoPER) analyzed in *ELK3*-depleted CUTLL1 cells using a Seahorse extracellular flux analyzer. (**E**) Heatmap presentation of glycolysis-related metabolites from metabolomic analysis in KOPTK1 cells (*n* = 3 per group). (**F**) qPCR analysis of glycolytic gene expression in *ELK3*-depleted KOPTK1 cells. (**G**) Density plot of ELK3 (red) and H3K27ac (blue) (GSE76783) occupancy around the transcription start site (TSS) of genes significantly down-regulated in ELK3-depleted KOPTK1 cells. ChIP-seq signal normalized with input. (**H**) Genome browser tracks of ELK3 and H3K27ac ChIP-seq signals at the promoters of *PGK1* (top) and *PFKFB4* (bottom). (**I**) ChIP-qPCR validation of ELK3 and H3K27ac binding to *PGK1* and *PFKFB4* promoters in KOPTK1 cells. (**J**) qPCR analysis of glycolysis-related genes in *FTO*-depleted KOPTK1 cells with or without the overexpression of *ELK3.* (**K**) Glycolytic proton efflux rate (glycoPER) analyzed in *FTO*-depleted CUTLL1 cells with or without the overexpression of *ELK3*. Data are presented as means (±SD) of technical triplicates, and the experiments were independently repeated at least twice with the similar results [(D), (F), (I), (J), and (K)]. Data were analyzed by one-way ANOVA with Tukey’s multiple comparisons (J), two-way ANOVA with Bonferroni’s multiple comparisons (A), and unpaired two-tailed Student’s *t* test [(D), (F), and (I)]. *P < 0.05, **P < 0.01, and ***P < 0.001.

To explore the functional significance of *ELK3* as an *FTO* downstream effector, we knocked down *ELK3* in CUTLL1 and KOPTK1 cells, along with two primary T-ALL cells. *ELK3* depletion suppressed T-ALL cell growth, analogous to the phenotype of *FTO* deficiency (Fig. 5A and fig. S7A). We next conducted RNA-seq following *ELK3* knockdown in CUTLL1 and KOPTK1 cells. KEGG pathway analysis of significantly down-regulated genes revealed top five enriched pathways, with glycolysis showing the most remarkable enrichment in both cells (Fig. 5B and fig. S7B). Consistently, gene set enrichment analysis (GSEA) also showed significant enrichment of glycolysis hallmark genes that were decreased in *ELK3*-deficient cells (Fig. 5C and fig. S7C). Seahorse analysis confirmed that *ELK3* depletion significantly reduced both basal and compensatory glycolytic rates (Fig. 5D and fig. S7D). Moreover, a targeted metabolomics analysis of glycolysis metabolites validated the reduction of several intermediates, including glucose-1-phosphate, fructose-6-phosphate, 3-phospho-glyceric acid, and phosphoenolpyruvic acid, upon *ELK3* depletion (Fig. 5E). Consistently, *ELK3* depletion also down-regulated multiple genes encoding glycolytic enzymes (Fig. 5F), suggesting that *ELK3* acts as a key transcription factor orchestrating glycolysis regulation in T-ALL.

To determine ELK3 direct transcriptional targets, we performed chromatin immunoprecipitation sequencing (ChIP-seq) in KOPTK1 cells. In agreement with a previous report (*35*), the most prominent proportion (87.07%) of the peak signals located in the promoters and ELK3 preferentially occupied the CCGGAAG sequence (fig. S7, E and F). ChIP-seq analysis revealed a notable enrichment of ELK3 near the transcription start sites (TSS), accompanied with the signal of H3K27ac (*36*), the epigenetic hallmark of transcriptional activation (Fig. 5G). Genome browser tracks demonstrated that ELK3, along with H3K27ac, directly bound to the promoter regions of various glycolytic genes, including *PGK1* and *PFKFB4* (Fig. 5H). Gene-specific ChIP-qPCR validation further corroborated these findings (Fig. 5I and fig. S7, G and H). Ectopic expression of *ELK3* efficiently rescued the expression of glycolytic genes, as well as the glycolysis defect following *FTO* ablation (Fig. 5, J and K, and fig. S7, I and J). Our findings thus highlight the functional significance of *ELK3* as a downstream effector of FTO, driving T cell leukemogenesis by regulating glycolysis.

### *FTO* inhibitor Dac51 exhibits potent anti–T-ALL efficacy in vitro and in vivo

**Fig. 6.**
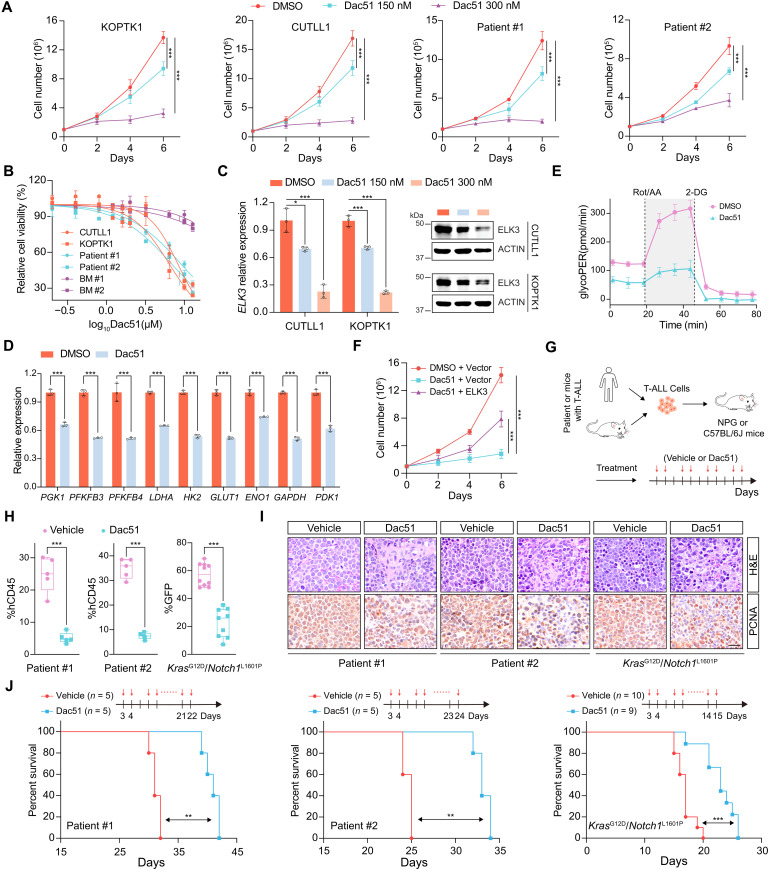
The FTO inhibitor Dac51 shows antileukemia efficacy in vitro and in vivo. (**A**) Growth curves of indicated T-ALL cells treated with varying doses of Dac51. (**B**) CCK8 viability assessment of various T-ALL cells and normal BM cells (BM #1 and #2) after Dac51 treatment for 24 hours. (**C**) qPCR (left) and immunoblots (right) showing the *ELK3* mRNA and protein levels upon Dac51 (150 or 300 nM) treatment for 36 hours. (**D**) qPCR analysis of glycolysis-related genes in CUTLL1 cells following Dac51 treatment (300 nM, 36 hours). (**E**) Assessment of glycolytic proton efflux rate (glycoPER) in KOPTK1 cells treated with 300 nM Dac51 for 36 hours. (**F**) Growth curves of 300 nM Dac51 treated CUTLL1 cells with or without ectopic *ELK3* expression. (**G**) Schematic of Dac51 treatment in vivo. Primary human or murine T-ALL cells were transplanted into NPG or WT C57BL/6J mice, respectively, followed by Dac51 (4 mg/kg) treatment. (**H**) Assessment of leukemia cells in the PB from T-ALL mice shown in (G). Percentages of hCD45^+^ leukemia cells were analyzed at day 20 post-transplantation (patient #1 and #2), and Kras^G12D^/Notch1^L1601P^ GFP^+^ murine leukemia cells were analyzed at day 14. (**I**) H&E and immunohistochemistry (PCNA) staining of spleens from T-ALL mice shown in (H) at 28, 20, and 14 days post-transplantation, respectively. Scale bar, 20 μm. (**J**) Kaplan-Meier survival curves of T-ALL mice shown in (H). Data are presented as the mean (±SD) from two biological replicates, each with three technical replicates [(A) and (F)], and from technical triplicates with experiments repeated at least twice [(B) to (E)]. Data were analyzed by one-way ANOVA with Tukey’s multiple comparisons (C), two-way ANOVA with Bonferroni’s multiple comparisons [(A) and (F)], unpaired two-tailed Student’s *t* test [(D) and (H)] and log-rank test (J). *P < 0.05, **P < 0.01, and ***P < 0.001.

We have thus far demonstrated that *FTO* is essential for the onset and maintenance of T-ALL. FTO inhibitors have shown remarkable antitumor effects (*26*, *37*, *38*). To test whether FTO serves as a therapeutic target in T-ALL, we treated various T-ALL cells with selective *FTO* inhibitors Dac51 or FB23-2 (*26*, *37*). As shown in Fig. 6A and fig. S8 (A and B), both compounds inhibited cell growth in a dose-dependent manner. Since Dac51 achieved more profound suppression, with 300 nM Dac51 producing an effect equivalent to 1500 nM FB23-2 (Fig. 6A and fig. S8, A to C), we chose Dac51 for further study. It is important to note that Dac51 induced profound lethal effects on various T-ALL cells while sparing normal BM cells (Fig. 6B), demonstrating its selective cytotoxicity toward transformed T cells.

Similar to *FTO* knockdown, Dac51 treatment decreased *ELK3* RNA and protein levels (Fig. 6C and fig.S8D), suppressed multiple glycolytic genes (Fig. 6D and fig. S8E), and inhibited glycolysis (Fig. 6E and fig. S8, F and G). Notably, enforced expression of *ELK3* antagonized Dac51-mediated growth defect (Fig. 6F and fig. S8, H and I), further corroborating the role of the FTO-ELK3 axis in promoting T-ALL cell growth.

To evaluate the antileukemia efficacy of Dac51 in vivo, we established patient-derived T-ALL xenografts (PDX) using primary T-ALL cells from various subtypes. Primary human T-ALL cells from two T-ALL subtypes (TAL1 and LMO2) were transplanted into NPG mice, respectively, followed by vehicle or Dac51 injection once per day (Fig. 6G). As shown in Fig. 6H, Dac51 treatment significantly reduced leukemia cell expansion in PB. H&E and immunohistochemical staining revealed fewer leukemia cell infiltration in the spleen following Dac51 treatment (Fig. 6I and fig. S8J). Consequently, administration of Dac51 substantially delayed T-ALL onset and prolonged the survival of recipient mice compared with the vehicle group (Fig. 6J and fig. S8K). We also set up allograft models using primary murine T-ALL cells harboring NOTCH1 mutation (L1601P) in the Kras^G12D^ genetic background (*39*) or overexpressing ICN1 alone. Similarly, Dac51 exhibited strong antileukemia activity in these animal models (Fig. 6 H to J, and fig. S8, J and K). In addition, minimal body weight differences were detected between treated or untreated groups (fig. S8 L). These in vitro and in vivo data provide strong evidence supporting Dac51 as a promising anti–T-ALL candidate drug with tolerable toxicity.

## DISCUSSION

**Fig. 7.**
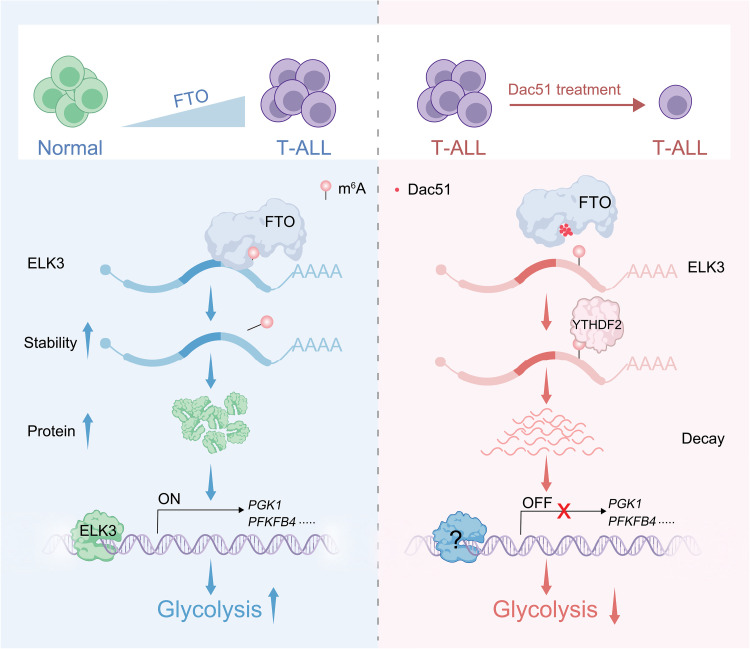
A working model depicting the crucial role of FTO and targeted strategy in T-ALL. Elevated FTO expression in T-ALL maintains low levels of m^6^A methylation on many transcripts including the *ELK3* mRNA, which prevents the YTHDF2-mediated degradation. Augmented ELK3 expression in turn up-regulates glycolysis-related genes like *PGK1* and *PFKFB4*, thereby enhancing glycolysis. Selective FTO inhibitor Dac51 leads to increased m^6^A modification on *ELK3* mRNA, resulting in decreased ELK3 expression, ultimately lowering glycolytic capacity and suppressing T-ALL progression. See more details in the text.

T-ALL is a heterogeneous disease resulting from a multistep transformation process in which genetic alterations in epigenetic regulators and chromatin modifiers are frequently found (*1*). Accumulating data demonstrate that aberrant activity or expression of these epigenetic factors plays a critical role in the initiation and development of T-ALL, leading to increasing number of small-molecule epigenetic drugs being evaluated in clinical trials (*40*). Despite large amount of work focuses on targeting epigenetic regulators that alter DNA methylation and post-translational modifications on histones, little is known about how the RNA modification and the regulators contribute to T-ALL progression. The RNA m^6^A modification represents a unique epigenetic mechanism in regulation of gene expression and a potential therapeutic target in human cancers, given that multiple m^6^A modifiers are deregulated during tumorigenesis (*10*, *41*). Both the m^6^A writers and erases are often found up-regulated in tumors as compared to normal tissue (*10*, *41*), suggesting that extraordinary m^6^A dynamics occurs in transformed cells. This is probably associated with swift epigenetic adaption of tumor cells to survive in arduous environment. Notably, the m^6^A demethylases FTO and ALKBH5 have been reported to be functionally essential in a variety of tumors such as AML and breast cancer (*42*, *43*). Disruption of either one leads to tumor growth suppression, most likely due to the regulation of respective key protumorigenic gene expression by each demethylase (*12*–*14*). We reveal that FTO, but not ALKBH5, is a unique dependency factor in T-ALL. Multi-omics studies and experimental validations identify transcriptional factor *ELK3* as a key and selective downstream effector regulated by FTO in an m^6^A-dependent manner. We further demonstrate ELK3 as a major transcription factor promoting glycolysis, providing additional mechanistic insights in molecular pathogenesis of T-ALL. These findings lay a theoretical foundation for therapeutic targeting of *FTO* in T-ALL (Fig. 7).

Whereas both m^6^A demethylases are more abundant in T-ALL in comparison to normal thymocytes, T-ALL onset and progression selectively depend on FTO. Although ALKBH5 may regulate m^6^A levels in T-ALL cells, the affected genes could be nonessential for the initiation and maintenance of T-ALL. The oncogenic or tumor-suppressive feature of demethylases may not be determined by overall m^6^A levels, but rather by m^6^A dynamics on specific key target genes driving leukemogenesis, such as *ELK3*, which is evidently not a shared target with ALKBH5. The differential dependency of FTO and ALKBH5 in the same tumor context has also been reported in bladder cancer (*44*, *45*) and B cell lymphoma (*46*). It is also interesting to note that ALKBH5, not the FTO, plays a crucial role in the function and development of T cells (*47*, *48*). We therefore reason that normal and transformed T cells exploit distinct m^6^A machinery to exert its physiological and pathological functions.

*ELK3* has been reported to act as an oncogene in various types of cancer (*30*) and reside in a risk locus for childhood ALL (*31*). However, the precise mechanism by which *ELK3* participates in tumorigenesis remains undefined. Our findings help decipher the oncogenic role of *ELK3* and the mechanism of action contributing to T-ALL progression. *ELK3* acts as a master regulator in controlling the expression of glycolysis genes by directly binding to their promoters. Intriguingly, *ELK3* was identified as a glucose-dependent gene in murine hypothalamic cells through analysis of the glucose-dependent transcriptome (*49*), suggesting a potential reciprocal regulation. FTO has been reported to directly regulate *PFKP* and *LDHB* expression to affect glycolysis in AML (*50*, *51*). Alternatively, we found that in T-ALL, FTO regulates glycolysis-related genes through ELK3. These data suggest that FTO reinforces glycolysis to drive leukemogenic program through multiple mechanisms and in a context-dependent manner. Metabolic rewiring in T-ALL is predominantly regulated by major transcriptional factors, such as NOTCH1, MYC, and RUNX2 (*52*–*54*), and our findings identify a previously unrecognized transcriptional factor ELK3 contributing to glycolytic capacity.

Our study thus highlights the indispensable role of FTO as m^6^A demethylase involved in glycolytic metabolism in T-ALL and provides a promising therapeutic strategy for T-ALL through the use of selective FTO inhibitor Dac51. Of note, FTO also mediates mRNA or snRNA m^6^A_m_ demethylation, in addition to m^6^A demethylation (*55*). Whether the m^6^A_m_ demethylase activity of *FTO* contributes to T-ALL progression remains unresolved. Considering that the overall abundance of m^6^A_m_ is significantly lower than that of m^6^A (*37*, *55*), the potent antileukemia efficacy triggered by Dac51 treatment is likely driven primarily by m^6^A demethylation. However, we cannot entirely rule out the possibility that FTO also exerts protumor effects through demethylation of m^6^A_m_. Given the multifunctional feature of FTO and its extraordinary protein stability (half-life >24 hours), designing agents that specifically degrade FTO, such as proteolysis-targeting chimeras (PROTAC), may offer a more effective strategy for achieving prolonged and durable antileukemia efficacy.

## MATERIALS AND METHODS

### Cell culture

T-ALL cell lines KOPTK1, CUTLL1, G4A2, and T6E were provided by W. Pear (University of Pennsylvania, USA). 293T cell line was purchased from American Type Culture Collection. Human T-ALL KOPTK1 and CUTLL1 cells, murine T-ALL G4A2, and T6E cells were cultured in RPIM-1640 with 10% fetal bovine serum (FBS; Hyclone), 1% penicillin-streptomycin (Gibco, 15140122), 1% nonessential amino acids (Gibco, 11140050), 2 mM l-glutamine (Gibco, 35050061), and 1 mM sodium pyruvate (Gibco, 11360070). 293T cells were cultured in DMEM (Hyclone) supplemented with 10% FBS (Gemini) and 1% penicillin-streptomycin. All cell lines are authenticated with short tandem repeats analysis and generally cultured at 37°C under 5% CO_2_ for fewer than 6 months after resuscitation and regularly tested for mycoplasma contamination using MycoAlert (Lonza).

Human BM cells were isolated from healthy donors and cultured in StemSpan SFEM II (StemCell Technologies, 09605) medium supplemented with rhSCF (100 ng/ml), rhFLT3L (100 ng/ml), rhTPO (50 ng/ml), and 1% penicillin-streptomycin. Primary human T-ALL cells used in this study were obtained from participants who provided informed written consent, and these cells were cocultured with MS5-DL1 feeder cells as previously described (*56*). All the cytokines were purchased from PeproTech. The study was conducted in accordance with protocols approved by the Ethics Committees of Soochow University (SUDA20201208H06) and Zhongnan Hospital of Wuhan University (2024063K).

### Mice

The *Alkbh5*^fl/fl^ and *Fto*^fl/fl^ mice were constructed as previously reported (*14*, *48*). *Lck*-Cre and *Mx1*-Cre strains were described as before (*25*). Both male and female mice at 8 to 10 weeks old were used for experiments, with age- and gender-matched littermates as control. NOD.Cg-Prkdc^scid^Il2rg^tm1Vst^/Vst (NPG) mice were purchased from Beijing Vitalstar. All mice were bred and maintained under specific pathogen–free conditions at the animal facility of Medical Research Institute at Wuhan University. All animal husbandry and experiments comply with the protocol approved by the Animal Care and Use Committee of Medical Research Institute (MRI2020-LAC40), Wuhan University.

### Plasmid construction

The shRNAs targeting human *FTO*, *ELK3*, or *YTHDF2* were cloned in pLKO.1. The coding regions FTO and ELK3 were amplified from the cDNA of KOPTK1 cells by PCR, and then cloned into the pCDH lentiviral vector. The enzymatic inactive mutant of FTO (H231A D233A) and the FTO construct that is resistant to the shFTO #1 by introducing silent mutations were generated using Q5 Site-Directed Mutagenesis Kit (NEB, E0554). For luciferase reporter assay, the WT or the mutant Exon4 of *ELK3* was inserted into pGL3-Promoter Vector (Promega) using ClonExpress Ultra One Step Cloning Kit (Vazyme, C115). *Escherichia coli* DH5α and Stbl3 *E. coli* were used in the transformation. shRNA targeting sequences are listed in table S2.

### Lentivirus preparation and infection

Lentiviruses from the pLKO.1 and pCDH-CMV-Pruo constructs were packaged with helper plasmid pMD2.G, psPAX2 (Addgene) in 293T cells. In brief, 9 μg of lentiviral constructs, 6 μg of psPAX2, and 3 μg of pMD2.G were cotransfected into 293T cells in 100-mm cell culture dish with Lipofectamine 2000 (Invitrogen, 11668019). Viral supernatants were generally collected 48 and 72 hours after transfection and concentrated with UltracelRegenerated Cellulose (Millipore). The appropriate amount of lentivirus was directly added into T-ALL cells in the presence of polybrene (8 μg/ml; Santa Cruz Biotechnology, sc-134220) and incubated at 37°C for 15 min, followed by centrifugation at 32°C, 1000*g* for 90 min. For cell lines, transduced cells were selected with puromycin (3 μg/ml; InvivoGen, ant-pr-1) 48 hours post-transduction. For primary T-ALL cells, GFP^+^ cells were isolated by flow cytometry 72 hours after transduction. These freshly transduced cells were subsequently prepared for functional analysis.

### RNA isolation and quantitative RT-PCR analysis

Total cellular RNA was extracted using TRIzol reagent (Takara, 9109). One microgram of RNA was used to synthesize cDNA, using the ReverTra Ace qPCR RT Kit according to the manufacturer’s instructions (TOYOBO, FSQ-101). qPCR was performed with ChamQ SYBR qPCR Master Mix (Vazyme, Q311-02) in CFX Connect Real-Time PCR System (Bio-Rad). Relative expression of the mRNA was calculated by the 2^−ΔΔCt^ method and normalized to ACTIN. All the specific PCR primer sequences are listed in table S2.

### Generation and analysis of murine ICN1-driven T-ALL model

Mouse BM transplantation was conducted as previously described (*25*). ICN1 expression retrovirus was generated by cotransfection of 9 μg of MigR1-ICN1 and 6 μg of packaging plasmid (pCgp and pHIT) into 293T cells in 100-mm cell culture dish with Lipofectamine 2000 (Invitrogen, 11668019). BM cells were collected from 6- to 8-week-old mice, and lineage negative (Lin^−^) cells were enriched with the Mouse Direct Lineage Cell Depletion Kit according to manufacturer’s instructions (Miltenyi Biotec, 130-110-470). The Lin^−^ cells were cultured in the presence of mTPO (50 ng/ml), mSCF (100 ng/ml), and mFLT3-L (50 ng/ml) and infected with concentrated MigR1-ICN retroviruses twice in the presence of polybrene (8 mg/ml; Santa Cruz Biotechnology, sc-134220). All the cytokines were purchased from PeproTech. GFP^+^ Lin^−^ cells (1 × 10^5^) were transplanted via tail vein injection into irradiated [6 grays (Gy)] 6- to 8-week-old WT C57BL/6 mice. For the T-ALL progression analysis shown in Fig. 2, all recipients were intraperitoneally injected with pIpC (10 mg/kg; InvivoGen, tlrl-pic-5) in physiological water (NaCl 0.9%) three times with a 1-day interval, while the GFP^+^ leukemia cells were detected after 3 weeks of transplantation. GFP^+^ leukemia cells in PB were analyzed periodically after transplantation.

### In vivo bioluminescence imaging

KOPTK1 cells were infected with pWPXLd-GFP-Luciferase lentivirus packaged with pMD2.G and psPAX2, and then GFP^+^ cells were sorted by BD FACS Aria III. GFP^+^ KOPTK1 cells were transduced with the appropriate lentivirus, and subsequently infected cells were subjected to selection using puromycin (3 μg/ml) to establish stable expression of luciferase. Seven- to 8-week-old NPG mice were irradiated (1.5 Gy) before tail vein injection of one million cells. For bioluminescence imaging, mice were given the substrate d-luciferin (150 mg/kg) by intraperitoneal injection, then anesthetized with isoflurane and imaged with Ami HTX (Spectral Instruments Imaging). The bioluminescent signals were quantified using Aura imaging software (Spectral Instruments Imaging).

### Dac51 treatment in PDX and murine allograft

To establish PDX model, primary T-ALL cells from patients were injected into the tail veins of 7- to 8-week-old irradiated (1.5 Gy) NPG mice at appropriate cell numbers: 1 × 10^5^ cells for patient #1 and 2 × 10^6^ cells for patient #2 and patient #3. For allograft model, 1 × 10^6^ GFP^+^ murine T-ALL cells driven by *Kras*^G12D^/*Notch1*^L1601P^ (*39*) or 1 × 10^5^ GFP^+^ ICN1-driven murine T-ALL cells were injected into the irradiated (4.5 Gy) WT C57BL/6J via tail injection. These mice were randomly grouped and treated with Dac51 (4 mg/kg per day) or vehicle control via daily intraperitoneal injection starting on day 3 post-transplantation.

### RNA-seq and data analysis

For RNA-seq, total RNA was extracted using TRIzol. Genomic DNA was removed by in-solution deoxyribonuclease (DNase) I treatment (Thermo Fisher Scientific, 89836). Poly(A) mRNA was subsequently purified from 1 μg of total RNA using Dynabeads mRNA purification kit (Invitrogen, 61006). RNA libraries were constructed using NEB Next Ultra Directional RNA Library Prep Kit (NEB, E7760) and sequenced as 150-bp paired-end reads by Illumina NovaSeq 6000 (Beijing Annoroad Co. Ltd). Sequencing quality was evaluated using FastQC and reads were mapped to human genome version GRCh38 by STAR (*57*). Differential expressed genes were analyzed by DESeq2 (*58*) and defined as |fold change| > 1.2 and *P* value <0.05 unless otherwise specified. We used KOBAS (*59*) for KEGG pathway analysis and used clusterProfiler (*60*) for GSEA analysis.

### m^6^A-seq and data analysis

For m^6^A-seq, m^6^A-IP was performed according to the protocol with some modifications (*61*). Total RNA was extracted from KOPTK1 cells using TRIzol reagent and then treated with DNase I (Thermo Fisher Scientific, 89836). mRNA was purified with Dynabeads mRNA Purification Kit (Invitrogen, 61006). One microgram of mRNA was fragmented into ~150 bp with RNA Fragmentation Reagents (Invitrogen, AM8740) at 90°C for 1 min. Twenty microliters of Dynabeads Protein A (Invitrogen, 10002D) was washed twice with IP buffer [10 mM tris-HCl (pH 7.5), 150 mM NaCl, and 0.5% Igepal CA-630] and mixed with 1 μg of m^6^A antibody (Abcam, ab151230) before orbital rotation at 4°C for 2 hours. The prepared mRNA was subsequently added for immunoprecipitation. The beads were washed twice with IP buffer, twice with low-salt reaction buffer [10 mM tris-HCl (pH 7.5), 50 mM NaCl, and 0.5% Igepal CA-630], and twice with high-salt reaction buffer [10 mM tris-HCl (pH 7.5), 500 mM NaCl, and 0.5% Igepal CA-630] before eluted with m^6^A elution buffer. The eluates and mRNA of input were purified by the RNA Clean & Concentrator-5 kit (Zymo Research, R1013) to construct libraries with the NEBNext Ultra Directional RNA Library Prep Kit (NEB, E7760) and sequenced as 150-bp paired-end reads by NovaSeq 6000 (Beijing Annoroad Co. Ltd). Reads were mapped to human genome version GRCh38 by STAR. m^6^A peaks were called by exomePeak version 2.16.0 (*62*), and 100-bp sequences around the peak center were extracted for motif enrichment with hypergeometric optimization of motif enrichment (HOMER) (*63*).

### Gene-specific m^6^A qPCR

m^6^A-qPCR was performed according to the m^6^A-seq procedure. Rabbit immunoglobulin G (IgG; Abclonal, AC005) was used as control. Purified mRNA fragments from IP and input samples were reverse transcribed using ReverTra Ace qPCR RT Kit, followed by qPCR with the primers listed in table S2.

### Metabolomics analysis

Targeted analysis of glycolysis metabolites was performed with the assistance of Shanghai Biotree Biotech according to a previous publication (*64*). In brief, ten million KOPTK1 cells were collected and quenched 30 s in liquid nitrogen. Metabolites were extracted using extraction solvent (*V*_methanol_:*V*_acetonitrile_:*V*_water_ = 2:2:1, −20°C) and dried in a vacuum concentrator. Metabolites were analyzed by TSQ Quantiva (Thermo Fisher Scientific) and quantified according to calibration curves.

### Chromatin immunoprecipitation sequencing

ChIP-seq was performed as previously with some modifications (*65*). Ten million KOPTK1 cells were cross-linked in phosphate-buffered saline (PBS) containing 1% formaldehyde (Sigma-Aldrich, F8775) for 10 min and then quenched with 0.125 M final concentration of glycine for 5 min at room temperature. Chromatins were sheared to an average size of ~300 bp using the Bruptor (Diagenode). Soluble chromatin containing supernatant was used for immunoprecipitation in IP buffer [10 mM tris-HCl (pH 8.0), 100 mM NaCl, and 1 mM EDTA] with the presence of 7 μg of ELK3 antibody (Novus, NBP1-83960) rotating overnight at 4°C. The DNA-antibody mixture was incubated with 30 μl of Protein A/G (Invitrogen, 80105G) for 4 hours at 4°C. The immunoprecipitate was washed three times with washing buffer I [20 mM tris-HCl (pH 8.0), 150 mM NaCl, 2 mM EDTA, 1% Triton X-100, and 0.1% SDS], washing buffer II [20 mM tris-HCl (pH 8.0), 500 mM NaCl, 2 mM EDTA, 1% Triton X-100, and 0.1% SDS], and washing buffer III [50 mM Hepes-KOH (pH 7.5), 1 mM EDTA, 250 Mm LiCl, 1% NP40, and 0.7% Na-deoxycholate], then eluted with 300 μl of elution buffer [50 mM tris-HCl (pH 8.0), 10 mM EDTA, and 1.0% SDS]. All buffers used were added with protease inhibitors. Cross-link reversal of immunoprecipitated DNA was performed overnight at 65°C and proteins were digested with 2 μl of proteinase K (Invitrogen, AM2546) at 55°C for 4 hours. DNA was purified using the phenol-chloroform extraction and ethanol precipitation.

Library preparation was done using the VAHTS Universal DNA Library Prep Kit for Illumina V4 (Vazyme, ND610-01), followed by sequencing on a NovaSeq 6000 (Beijing Annoroad Co. Ltd). Reads were aligned to the human genome (GRCh38) using bowtie2 (*66*). MACS2 was used to call peaks and an initial threshold *q* value of 0.01 was set as the cutoff (*67*). The IP signal was normalized to input and bigwig files were visualized in the IGV software. Peak annotation and motif analysis were performed on ChIPseeker and HOMER, respectively (*63*, *68*).

### m^6^A dot blot assay

m^6^A dot blot assays were performed as described previously with minor modifications (*12*, *46*). In brief, total RNA was extracted from cell pellets using TRIzol. The concentration of the total RNA sample was determined by Nanodrop 2000 (Thermo Fisher Scientific). Indicated amounts of total RNA were denatured at 65°C for 5 min before samples were loaded onto the Amersham Hybond-N+ membrane (GE Healthcare, RPN303B). After cross-linking under 254-nm ultraviolet for 2 min, membranes were washed for 5 min at room temperature, blocked in 5% milk in PBS with Tween 20 (PBST) buffer for 1 hour, and then incubated with the m^6^A antibody (CST, 56593S) at 4°C overnight. The membranes were washed three times with PBST and incubated with horseradish peroxidase (HRP)–conjugated goat antirabbit IgG (Jackson ImmunoResearch, JAC-111-035-003) for 1 hour at room temperature before developing with Clarity Western ECL Substrate (Bio-Rad, 1705061) and imaged on the Chemidoc Touch imaging system (BioRad). Equal loading of RNA was confirmed by staining of the membrane with 0.02% methylene blue in 0.3 M sodium acetate (pH 5.2).

### RNA immunoprecipitation

Ten million cells were collected and washed with ice-cold PBS before resuspended in 1.1 ml of RIP lysis buffer [50 mM tris-HCl (pH 7.0), 150 mM NaCl, 2 mM EDTA, and 0.5% Igepal CA-630], containing RNasin (100 U/ml; Promega, N2111) and protease inhibitors (Roche, 04693159001). Lysates were centrifuged at 4°C to remove cell debris after incubating on ice for 30 min. Supernatants were collected into a new tube and 50 μl was kept for input control. The remaining lysates were divided into equal volumes (500 μl) to incubate with either 4 μg of antibody (Proteintech; FTO, 27226-1-AP; YTHDF2, 24744-1-AP) or rabbit IgG (Abclonal, AC005) rotating overnight at 4°C. Thirty microliters of Dynabeads Protein G (Invitrogen, 10004D), prewashed three times with RIP lysis buffer, was added to the samples and rotated at 4°C for 4 hours. Beads were washed with RIP lysis buffer five times before RNA was purified by TRIzol. cDNA synthesis was performed using the ReverTra Ace qPCR RT Kit according to the manufacturer’s instructions. qPCR was used to measure bound RNA abundance.

### RNA stability assay

For RNA stability assay, two million cells were treated with actinomycin D (Sigma-Aldrich, A9415) at a final 5 μg/ml concentration and collected at indicated time points. Total RNA was extracted, reverse transcribed, and used for reverse transcriptase qPCR. ACTIN was used as endogenous control. mRNA’s degradation rate was estimated according to the previously published paper (*69*).

### Dual-luciferase reporter and mutagenesis assays

The WT or mutant ELK3-Exon4 was subcloned into the downstream of firefly luciferase of pGL3-Promoter Vector in-frame (Promega). For dual-luciferase reporter assay, 400 ng of WT or mutant ELK3-Exon4 constructs and 20 ng of pRL-TK Renilla were cotransfected into 293T cells using Lipofectamine 2000 (Invitrogen, 11668019). Luciferase activities were measured 24 hours later using Dual-Luciferase Reporter Assay System (Promega, E1960). The relative luciferase activities were normalized to Renilla luciferase control under the same treatment conditions.

### Seahorse extracellular flux analysis

Glycolytic capacity of T-ALL cells was measured as their glycolytic proton efflux rate (glycoPER) with Seahorse XFe24 Extracellular Flux Analyzer and a Seahorse XF Glycolytic Rate Assay Kit (Agilent, 103344-100) according to the manufacturer’s instruction. T-ALL cells (1 to 1.5 × 10^5^) per well were plated in XFe24 cell culture microplates precoated with Cell-Tak (22.4 μg/ml; Corning, 354240). The cells were then incubated in Seahorse XF RPMI medium supplemented with 2 mM l-glutamine, 1 mM sodium pyruvate and 10 mM d-glucose (Agilent, 103681-100) for 45 min in CO_2_-free incubator before the assay. Data were analyzed using Wave software and Seahorse Glycolytic Rate Assay Report Generator (Agilent).

### Measurement of glucose uptake and lactate secretion

Glucose uptake or lactate secretion was measured using respective Colorimetric Assay Kits (ab136955, K6027-100, BioVision). In brief, 1 × 10^6^ cells were seeded at 12-well plates and culture medium were then collected to quantify glucose and lactate according to the manufacturer’s instructions. The values of consumed glucose and released lactate were normalized to the same total protein.

### Immunoblotting

Proteins were extracted using RIPA buffer [50 mM tris-HCl (pH 7.4), 150 mM NaCl, 1 mM EDTA, 0.1% SDS, 1% sodium deoxycholate, and 1% NP-40], supplemented with a protease inhibitor cocktail (Roche, 4693132001) and protein concentrations were determined using BCA assay kit (ZOMANBIO, ZD301). Equal amounts of protein were fractionated by various concentrations of SDS–polyacrylamide gel electrophoresis and transferred to a polyvinylidene difluoride membrane (Bio-Rad). Membranes were blocked for 1 hour at room temperature in TBST supplemented with 5% nonfat dried milk and incubated overnight at 4°C with primary antibody diluted in the same blocking buffer. After washing three times in TBST, membranes were incubated for 1 hour at room temperature with appropriate HRP-conjugated secondary antibodies (Jackson ImmunoResearch JAC-111-035-003 or JAC-115-035-003). The signal was detected using ECL Western Blotting Substrate (Bio-Rad) on the Chemidoc Touch imaging system (Bio-Rad). Antibodies used in the experiments include FTO (Proteintech, 27226-1-AP), ELK3 (OriGene, TA503603; NOVUS, NBP2-01264), ALKBH5 (Abcam, ab195377), and β-ACTIN (Abclonal, AC026).

### H&E staining and immunohistochemistry

The tissue samples were fixed in a 4% formaldehyde solution, followed by processing and embedding in paraffin wax. Serial sections (3 μm in thickness) were obtained and subjected to H&E staining (C0105, Beyotime). For immunohistochemical analysis, paraffin-embedded spleen sections were incubated with a PCNA antibody (Santa Cruz Biotechnology, sc-56) at a 1:200 dilution. Staining results were visualized using the DAB substrate kit from Vector Laboratories, and images were captured using CaseViewer (3DHISTECH).

### Wright-Giemsa staining

PB obtained from T-ALL mice was diluted with an equal volume of PBS and then subjected to a smear. Following air drying, the slides were incubated in Giemsa stain for 30 s and subsequently stopped with buffer B following the manufacturer’s instructions (BaSO, BA4017). After a gentle rinse with ddH_2_O, the slides were dried for imaging using Aperio VERSA 8 (Leica).

### Flow cytometry analysis

Percentage of GFP^+^ leukemia in the PB or spleen of T-ALL mice was analyzed by Accuri C6 (BD Biosciences) or CytoFLEX (Beckman) after excluding red blood cells using Red Blood Cell Lysis Buffer (BioLegend, 420301). Live cells were gated on the basis of FSC-A and SSC-A characteristics.

### Statistical analysis

All quantitative data are presented as means ± SD. Statistical analyses were performed using GraphPad Prism8 and R software. Statistical analyses were performed using two-tailed, unpaired Student’s *t* test, Mann-Whitney test, or Welch’s *t* test for comparisons between two groups, while one-way analysis of variance (ANOVA) with Tukey’s multiple comparisons or two-way ANOVA with Bonferroni’s multiple comparisons was used for experiments involving multiple groups. Significance of Kaplan-Meier survival curves was estimated by log-rank test. Unless otherwise stated in the text, differences were considered significant when *P* < 0.05.
