# Supplementary material for: FTO regulates ELK3-mediated metabolic rewiring and represents a unique therapeutic target in T cell leukemia
Source: Sci Adv. 2025 May 28;11(22):eadq3052. doi: 10.1126/sciadv.adq3052 (PMC12118595; doi:10.1126/sciadv.adq3052)
Supplement: Supplementary file 1 — Figs. S1 to S8 Tables S1 and S2 [file sciadv.adq3052_sm.pdf]

Science Advances
AAAS

Supplementary Materials for

FTO regulates ELK3-mediated metabolic rewiring and represents a unique therapeutic target in T cell leukemia

Hao Huang *et al.*

Corresponding author: Hudan Liu, hudanliu@whu.edu.cn; Guoliang Qing, qingguoliang@whu.edu.cn; Yun Zhao, zhaoy@suda.edu.cn

Sci. Adv. **11**, eadq3052 (2025)
DOI: 10.1126/sciadv.adq3052

This PDF file includes:

Figs. S1 to S8
Tables S1 and S2

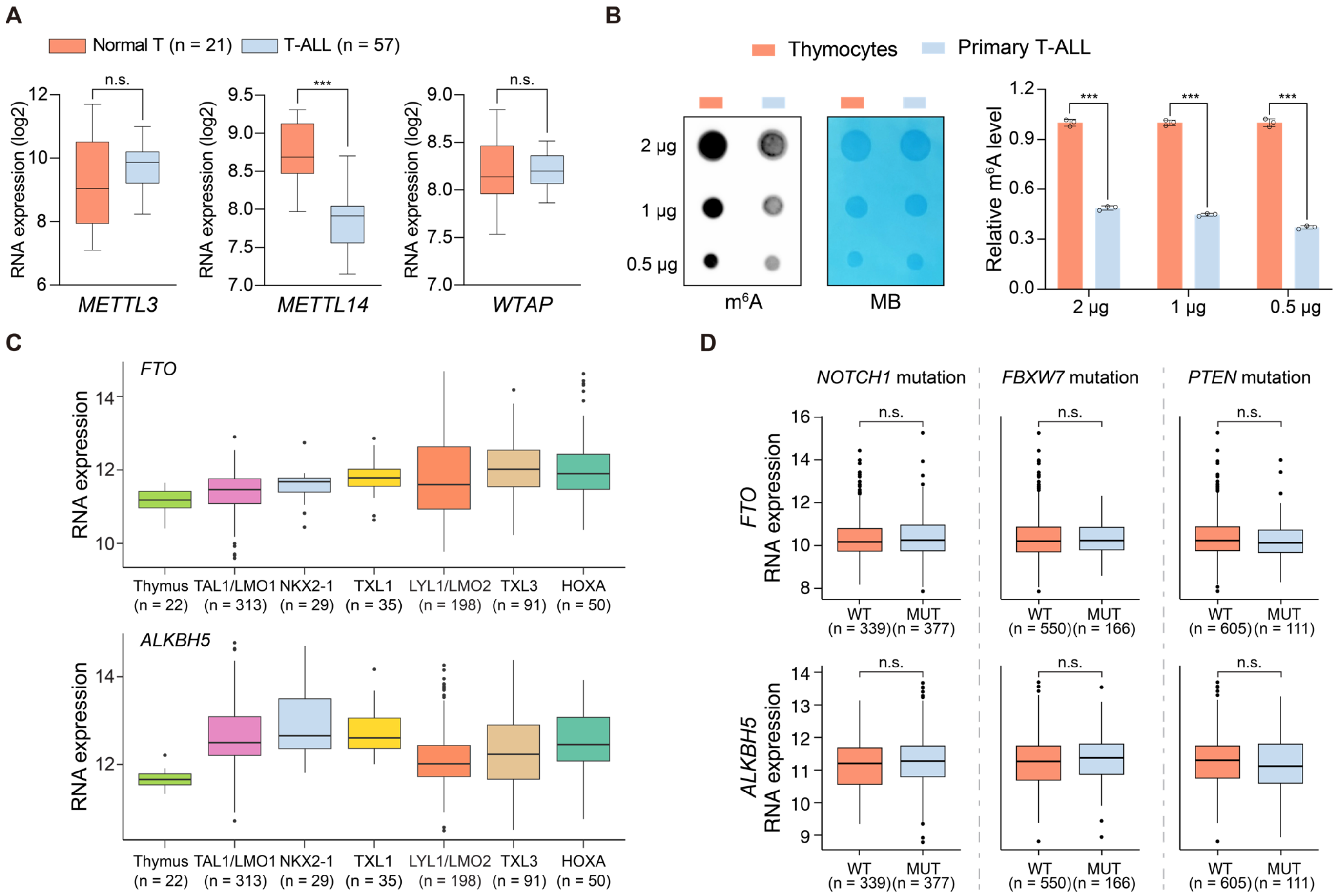

Fig. S1. Elevated *FTO* and *ALKBH5* expression in T-ALL.

(**A**) Expression analysis of *METTL3*, *METTL14*, and *WTAP* in 57 primary T-ALL patients and 21 normal T cells, similar to Fig. 1A.

(**B**) Representative image (left) and quantification (right) of the m^{6}A dot blot showing global m^{6}A abundance in murine thymocytes and primary murine T-ALL cells. Methylene blue (MB) staining was used as a loading control.

(**C-D**) Analysis of *FTO* and *ALKBH5* mRNA expression in 716 primary T-ALL samples categorized into various subgroups (C), or grouped by differential *NOTCH1*, *FBXW7*, and *PTEN* mutational status (D). 22 normal thymocyte samples were used as control. Data are presented as mean (± SD). Statistical significance was determined by Welch t-test (A, D) and unpaired two-tailed Student's t-test (B). ***$P < 0.001$, n.s., non-significant

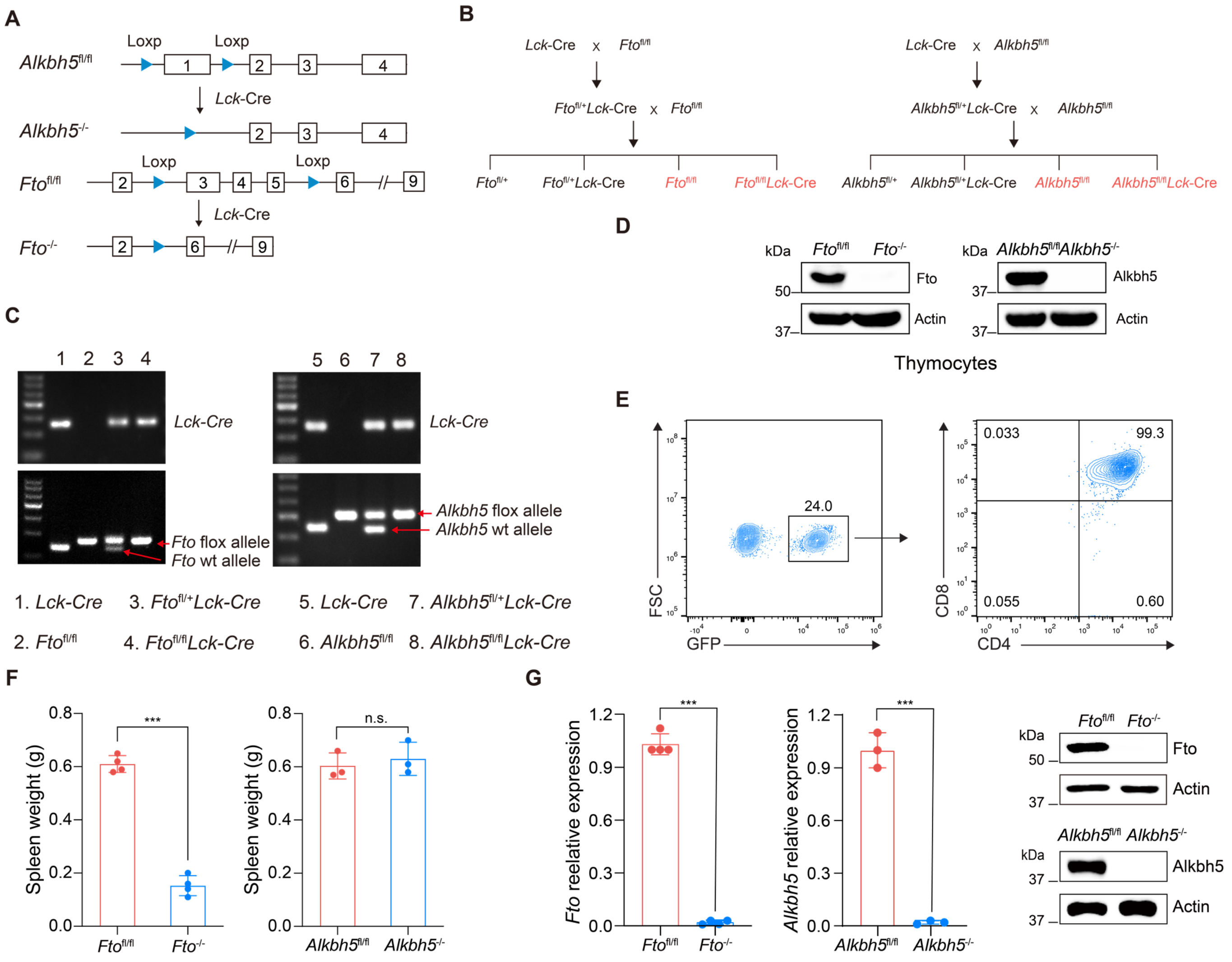

Fig. S2. Generation of *Fto* and *Alkbh5* conditional knockout mice.

(**A-B**) Scheme for generating *Fto*$^{fl/fl}$, *Fto*$^{-/-}$, *Alkbh5*$^{fl/fl}$, and *Alkbh5*$^{-/-}$ mouse.

(**C**) Representative DNA genotyping image of indicated mice.

(**D**) Immunoblot showing the depletion of *Fto* and *Alkbh5* in murine thymocytes.

(**E**) Representative flow cytometry images showing the CD4 and CD8 immunophenotype of GFP^{+} leukemia cells detected in Fig. 1C.

(**F**) Spleen weights of *Fto*$^{-/-}$ (n = 4 per group) or *Alkbh5*$^{-/-}$ (n = 3 per group) transplant mice in Fig. 1F.

(**G**) mRNA (left) and protein levels (right) of *Fto* (n = 4 per group) or *Alkbh5* (n = 3 per group) were analyzed by qPCR and immunoblot in primary murine leukemia cells, respectively.

Data are presented as mean (± SD). Statistical significance was determined by unpaired two-tailed Student's t-test (F, G). ****P* < 0.001, n.s., non-significant.

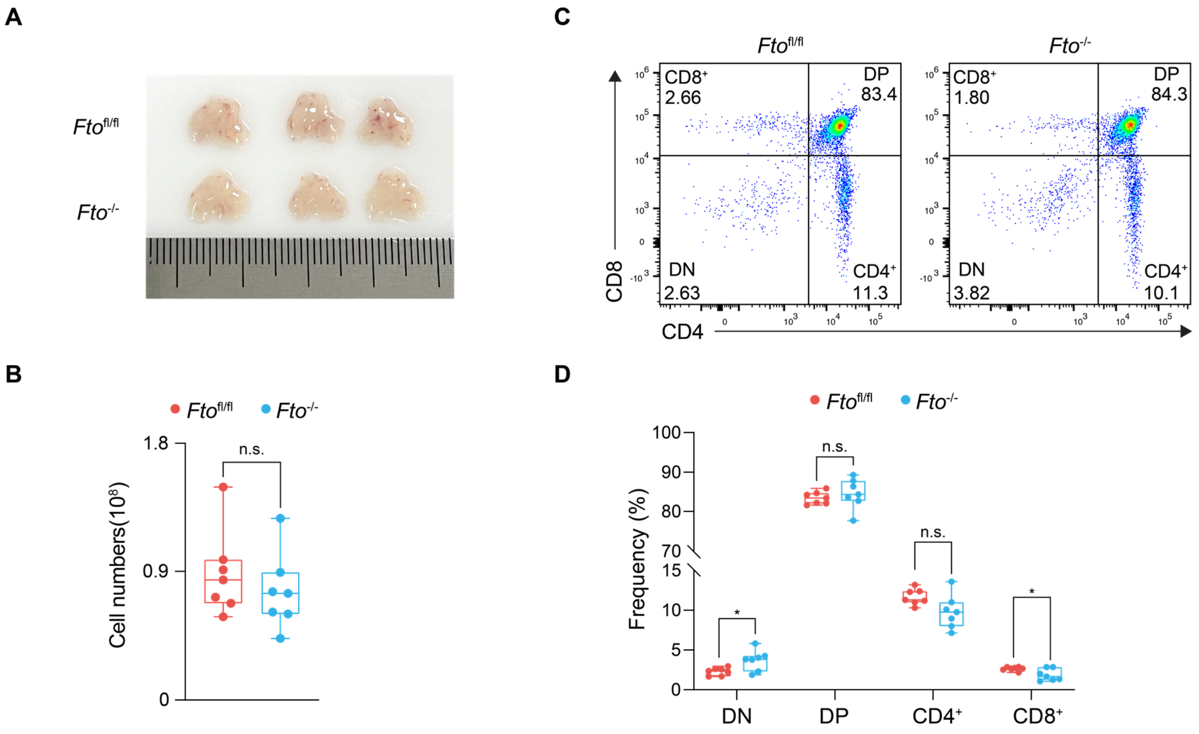

Fig. S3. *Fto* knockout shows minimal effect on thymic development.

(A-B) Thymus morphology (A) and total cell numbers of thymocytes (B) from 7-week-old *Fto*$^{fl/fl}$ and *Fto*$^{-/-}$ mice (n = 7 per group).

(**C-D**) Representative flow cytometry analysis of thymocytes stained for CD4 and CD8 (C), with corresponding statistical analysis of their frequencies (D) (n = 7 per group). DN, CD4$^-$CD8$^-$ double negative. DP, CD4$^+$CD8$^+$ double positive. Data are presented as mean (± SD). Statistical significance was determined by Welch t-test. *$P < 0.05$, n.s., non-significant.

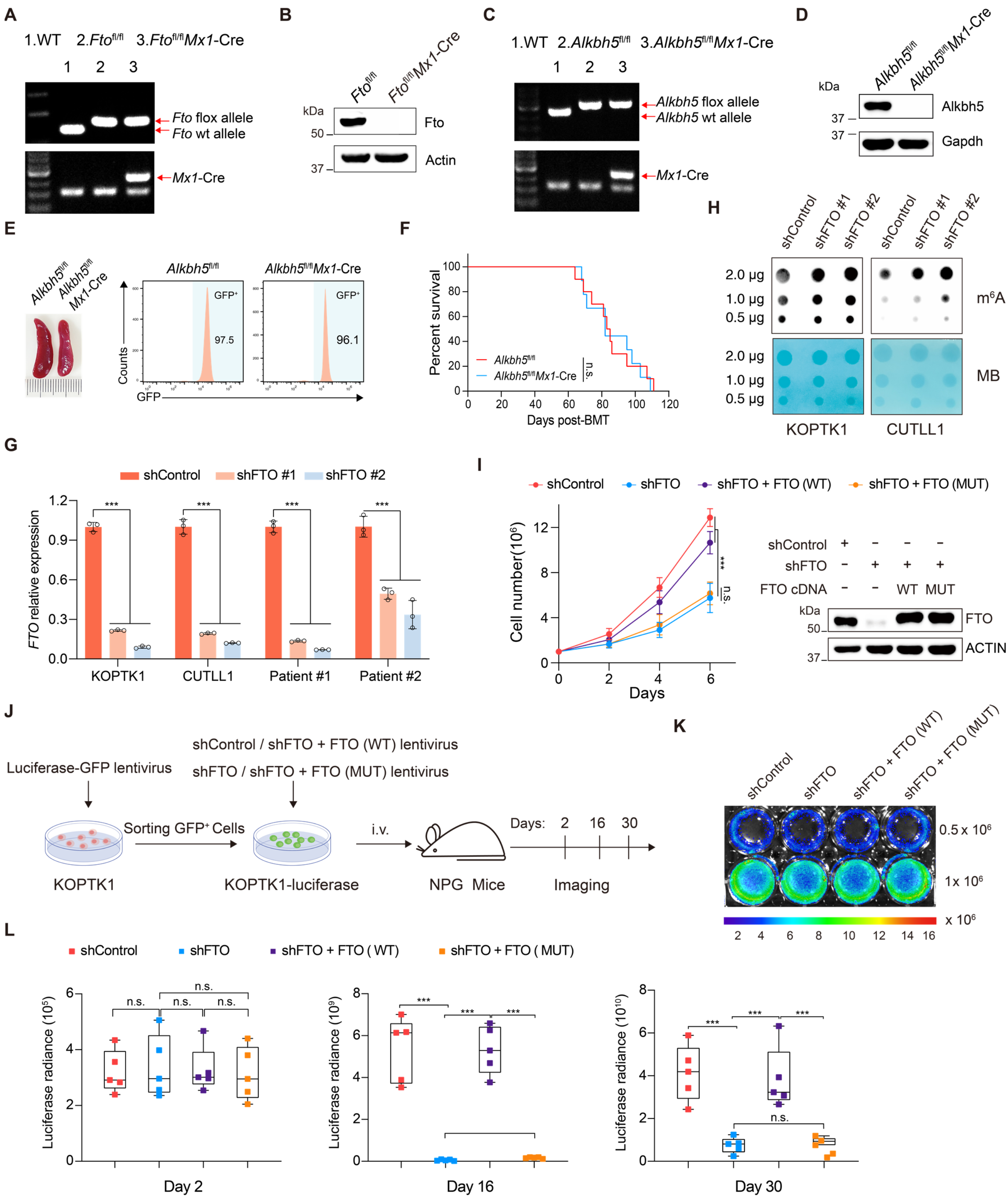

A
1.WT 2.Fto fl/fl 3.Fto fl/fl Mx1-Cre
Fto flox allele
Fto wt allele
Mx1-Cre
B
Fto fl/fl
Fto fl/fl Mx1-Cre
kDa
Fto
Actin
C
1.WT 2.Alkbh5 fl/fl 3.Alkbh5 fl/fl Mx1-Cre
Alkbh5 flox allele
Alkbh5 wt allele
Mx1-Cre
D
Alkbh5 fl/fl
Alkbh5 fl/fl Mx1-Cre
Alkbh5
Gapdh
E
Counts
GFP
97.5
96.1
F
Percent survival
Days post-BMT
n.s.
H
shControl
shFTO #1
shFTO #2
2.0 μg
1.0 μg
0.5 μg
m6A
MB
KOPTK1
CUTLL1
G
FTO relative expression
KOPTK1
CUTLL1
Patient #1
Patient #2
I
shControl
shFTO
shFTO + FTO (WT)
shFTO + FTO (MUT)
Cell number(10^6)
Days
FTO cDNA
WT MUT
FTO
ACTIN
J
Luciferase-GFP lentivirus
shControl / shFTO + FTO (WT) lentivirus
shFTO / shFTO + FTO (MUT) lentivirus
Sorting GFP+ Cells
i.v.
KOPTK1
KOPTK1-luciferase
NPG Mice
Days: 2 16 30
Imaging
K
0.5 x 10^6
1x 10^6
x 10^6
L
Luciferase radiance (10^5)
Luciferase radiance (10^9)
Luciferase radiance (10^10)
Day 2
Day 16
Day 30

Fig. S4. *FTO* promotes T-ALL progression in an m^{6}A-dependent manner.

(**A**) Representative DNA genotyping image of wild type (WT), *Fto*$^{fl/fl}$ and *Fto*$^{fl/fl}$*Mx1*-Cre mice.

(**B**) Immunoblots showing Fto protein expression in murine leukemia cells from recipient mice following pIpC injection. Leukemia cells were collected at the time of T-ALL induced mortality.

(**C**) Representative DNA genotyping image of wild-type (WT), *Alkbh5*$^{fl/fl}$ and *Alkbh5*$^{fl/fl}$ *Mx1*-Cre mice.

(**D**) Immunoblots showing the expression of *Alkbh5* protein in murine leukemia cells from recipient mice after pIpC injection. Leukemia cells were collected at the time of T-ALL induced mortality.

(**E**) Representative spleen images from indicated recipient mice 8 weeks post-engraftment.

(**F**) Kaplan-Meier curves of recipient mice transplanted with ICN1-transduced BM Lin$^-$ cells from *Alkbh5*$^{fl/fl}$ (n = 10) and *Alkbh5*$^{fl/fl}$ *Mx1*-Cre mice (n = 9).

(**G**) qPCR analysis of *FTO* in T-ALL cells with or without *FTO* knockdown.

(**H**) m^{6}A dot blot assay assessing the global m^{6}A level after *FTO* knockdown in T-ALL cells. Methylene blue (MB) staining was used as the loading control.

(**I**) Growth curves of CUTLL1 cells upon *FTO* depletion, with or without the restoration of wild-type (WT) or m^{6}A demethylase-inactive mutant (MUT) FTO (left). Data are presented as means (±SD) from two independent biological replicates, each performed with three technical replicates.

(**J**) Experimental scheme of *in vivo* imaging of KOPTK1-luciferase human T-ALL xenograft showing in Fig. 2H. KOPTK1-luciferase cells expressing luciferase and green fluorescent protein (GFP) transduced with indicated lentiviruses were then injected into irradiated NPG mice, monitoring tumor burden as stated time.

(**K**) Representative image showing luciferase luminescence signals across different cell numbers of KOPTK1-luciferase cells transduced with the indicated lentivirus to ensure equivalent starting signals.

(**L**) Quantification of the luciferase luminescence signals as observed in Fig. 2H.

Data are presented as means (±SD) of technical triplicates, and the experiments were independently repeated twice with the similar results (G). Data were analyzed by one-way ANOVA with Tukey's multiple comparisons (G, L) and two-way ANOVA with Bonferroni's multiple comparisons (I). ***$P < 0.001$, n.s., non-significant.

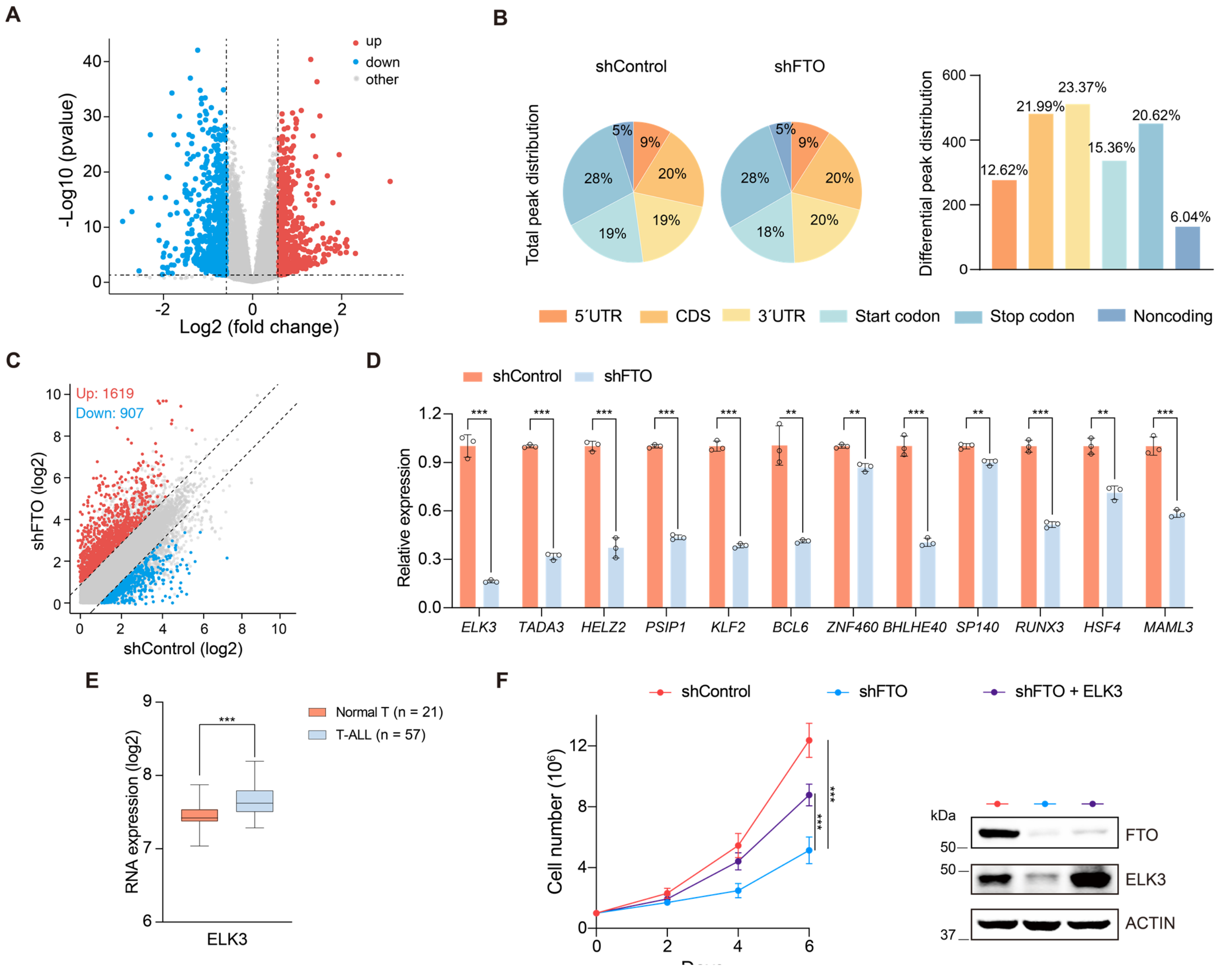

Fig. S5. Identification of FTO potential targets in T-ALL cells.

(**A**) Volcano plot of differentially expressed genes upon *FTO* depletion in KOPTK1 cells, related to Fig. 3A.

(**B**) The distribution of total m^{6}A peaks (left) or differential m^{6}A peaks (right) upon *FTO* knockdown in six transcript segments.

(**C**) Scatter plots showing the m^{6}A enrichment of identified m^{6}A peaks with significantly increased (red) or decreased (blue) in *FTO* depleted KOPTK1 cells compared with control cells.

(**D**) qPCR analysis of genes encoding transcription factors in *FTO* depleted KOPTK1 cells. Data are presented as means (±SD) of technical triplicates, and the experiments were independently repeated twice with the similar results.

(**E**) Expression analysis of *ELK3* among 57 primary T-ALL patients and 21 normal T cells. Datasets used are shown in Fig. 1A.

(**F**) Growth curves of *FTO* depleted CUTLL1 cells with or without the ectopic expression of *ELK3* (left). Immunoblots of FTO and ELK3 are shown on the right. Data are presented as means (±SD) from two independent biological replicates, each performed with three technical replicates.

Data were analyzed by unpaired two-tailed Student's t-test (D), Welch t-test (E) and two-way ANOVA with Bonferroni's multiple comparisons (F). $^{**}P < 0.01$ and $^{***}P < 0.001$.

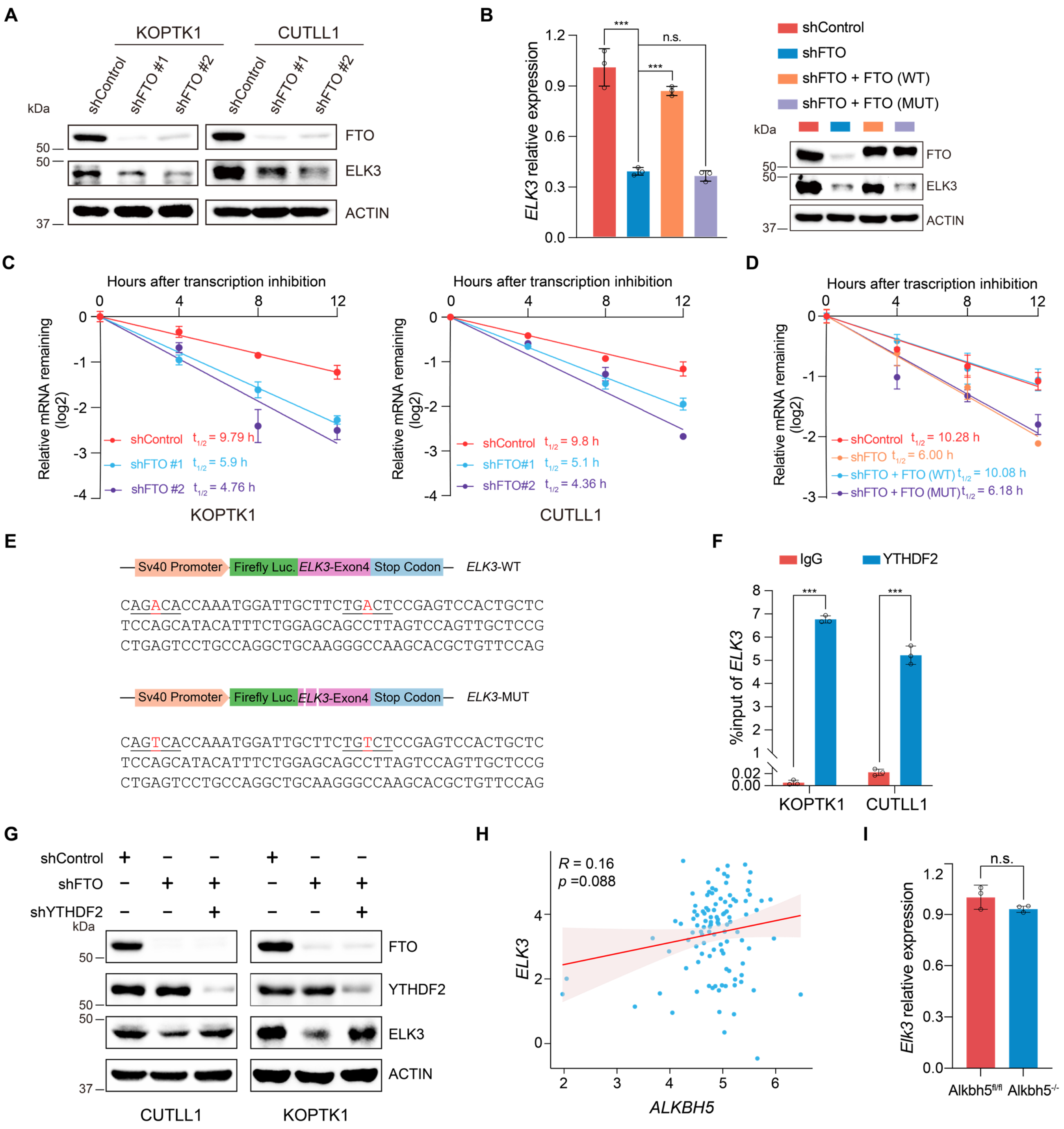
A
KOPTK1
CUTLL1
shControl
shFTO #1
shFTO #2
kDa
50
50
37
FTO
ELK3
ACTIN
B
ELK3 relative expression
1.2
0.9
0.6
0.3
0.0
***
n.s.
shControl
shFTO
shFTO + FTO (WT)
shFTO + FTO (MUT)
C
Hours after transcription inhibition
0
4
8
12
Relative mRNA remaining (log2)
shControl t1/2 = 9.79 h
shFTO #1 t1/2 = 5.9 h
shFTO #2 t1/2 = 4.76 h
KOPTK1
shControl t1/2 = 9.8 h
shFTO#1 t1/2 = 5.1 h
shFTO#2 t1/2 = 4.36 h
CUTLL1
D
Hours after trascription inhibition
shControl t1/2 = 10.28 h
shFTO t1/2 = 6.00 h
shFTO + FTO (WT) t1/2 = 10.08 h
shFTO + FTO (MUT) t1/2 = 6.18 h
E
Sv40 Promoter
Firefly Luc.
ELK3-Exon4
Stop Codon
ELK3-WT
CAGACACCAAATGGATTGCTTCTGACTCCGAGTCCACTGCTC
TCCAGCATACATTTCTGGAGCAGCCTTAGTCCAGTTGCTCCG
CTGAGTCCTGCCAGGCTGCAAGGGCCAAGCACGCTGTTCCAG
ELK3-MUT
CAGTCACCAAATGGATTGCTTCTGTCTCCGAGTCCACTGCTC
TCCAGCATACATTTCTGGAGCAGCCTTAGTCCAGTTGCTCCG
CTGAGTCCTGCCAGGCTGCAAGGGCCAAGCACGCTGTTCCAG
F
IgG
YTHDF2
%input of ELK3
8
7
6
5
4
3
2
1
0.02
0.00
KOPTK1
CUTLL1
G
shControl
shFTO
shYTHDF2
FTO
YTHDF2
ELK3
ACTIN
CUTLL1
KOPTK1
H
R = 0.16
p =0.088
ELK3
ALKBH5
I
n.s.
Elk3 relative expression
Alkbh5fl/fl
Alkbh5-/-

Fig. S6. *FTO* regulates *ELK3* expression through enhancing its mRNA stability.
(**A**) Immunoblots showing protein level of *ELK3* using two different shRNA knockdown *FTO* in leukemia cells.
(**B**) qPCR (left) and immunoblot (right) analysis of *ELK3* expression in *FTO* depleted CUTLL1 cells rescued with WT or MUT FTO.
(**C**) The mRNA half-life ($t_{1/2}$) of *ELK3* in *FTO* knockdown T-ALL cells.
(**D**) The mRNA half-life ($t_{1/2}$) of *ELK3* in *FTO* knockdown CUTLL1 cells with enforced expression of WT or MUT FTO.
(**E**) DNA sequence of WT or MUT exon4 of *ELK3* used in Fig. 4H. Potential m^6A sequences are underlined and the mutant bases are highlighted in red.
(**F**) YTHDF2-RIP qPCR showing binding of YTHDF2 to ELK3 mRNA in T-ALL cells.
(**G**) Immunoblots showing *ELK3* protein levels in *FTO*-deficient T-ALL cells with or without YTHDF2 shRNA expression.
(**H**) Scatterplot showing the correlation of *ALKBH5* and *ELK3* in 108 T-ALL samples from databases of National Omics Data Encyclopedia (NODE) (OEP002748). The expression levels were $\log_2$(TPM). Pearson correlation coefficient (R) and p-value (p) are marked.
(**I**) mRNA levels of *Elk3* in primary murine T-ALL cells from *Alkbh5*$^{fl/fl}$ or *Alkbh5*$^{-/-}$ BMT as shown in Fig. S2G (n = 3 per group).
Data are presented as means (±SD) of technical triplicates, and the experiments were independently repeated at least twice with the similar results (B-D, F). Data were analyzed by one-way ANOVA with Tukey's multiple comparisons (B) and unpaired two-tailed Student's t-test (F, I). ns, non-significant. ***$P < 0.001$, n.s., non-significant.

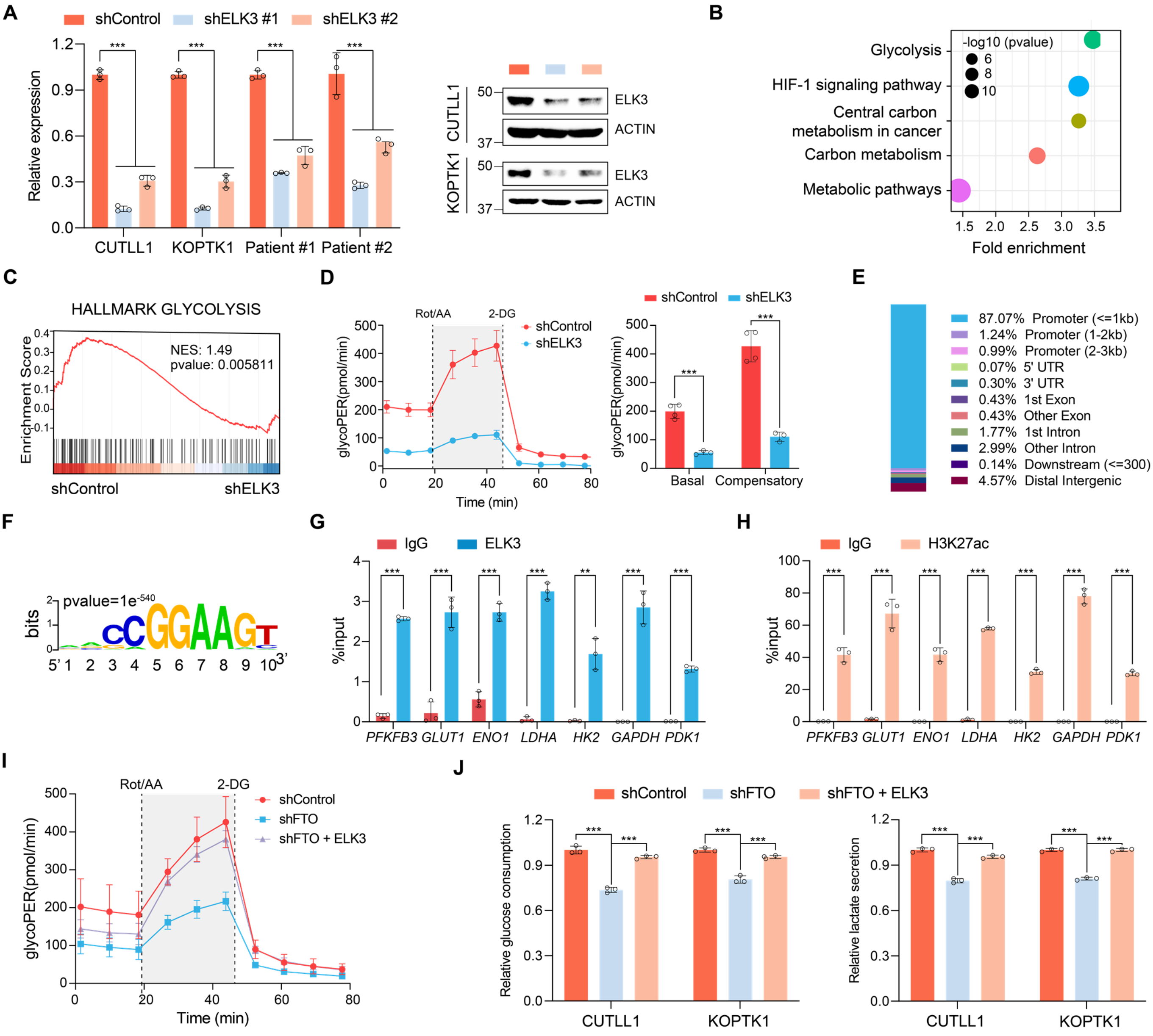
A
shControl
shELK3 #1
shELK3 #2
Relative expression
1.2
0.9
0.6
0.3
0.0
***
CUTLL1
KOPTK1
Patient #1
Patient #2
CUTLL1
KOPTK1
50
37
ELK3
ACTIN
B
Glycolysis
HIF-1 signaling pathway
Central carbon metabolism in cancer
Carbon metabolism
Metabolic pathways
-log10 (pvalue)
6
8
10
1.5 2.0 2.5 3.0 3.5
Fold enrichment
C
HALLMARK GLYCOLYSIS
Enrichment Score
NES: 1.49
pvalue: 0.005811
shControl
shELK3
D
Rot/AA
2-DG
shControl
shELK3
glycoPER(pmol/min)
Time (min)
Basal
Compensatory
E
87.07% Promoter (<=1kb)
1.24% Promoter (1-2kb)
0.99% Promoter (2-3kb)
0.07% 5' UTR
0.30% 3' UTR
0.43% 1st Exon
0.43% Other Exon
1.77% 1st Intron
2.99% Other Intron
0.14% Downstream (<=300)
4.57% Distal Intergenic
F
pvalue=1e-540
bits
5' 1 2 3 4 5 6 7 8 9 10 3'
G
IgG
ELK3
%input
PFKFB3 GLUT1 ENO1 LDHA HK2 GAPDH PDK1
H
IgG
H3K27ac
%input
PFKFB3 GLUT1 ENO1 LDHA HK2 GAPDH PDK1
I
Rot/AA
2-DG
shControl
shFTO
shFTO + ELK3
glycoPER(pmol/min)
Time (min)
J
shControl
shFTO
shFTO + ELK3
Relative glucose consumption
Relative lactate secretion
CUTLL1
KOPTK1

Fig. S7. ELK3 acts as a functionally important target of FTO through regulating glycolysis.

(**A**) qPCR (left) and immunoblots (right) showing the *ELK3* mRNA and protein levels using two different shRNA in T-ALL cells.

(**B**) KEGG pathway analysis of genes showing significant downregulation in *ELK3* depleted CUTLL1 cells.

(**C**) GSEA plot showing enrichment of the hallmark glycolysis gene set in *ELK3* knockdown KOPTK1 cells.

(**D**) Glycolytic proton efflux rate (glycoPER) analyzed in *ELK3* depleted KOPTK1 cells using a Seahorse extracellular flux analyzer.

(**E**) Categories of ELK3 ChIP-seq peaks as depicted.

(**F**) Top motif was identified in ELK3 ChIP-seq by HOMER. p-value was calculated with the hypergeometric test.

(**G-H**) ChIP-qPCR showing the binding of ELK3 (G) or H3K27ac (H) to the promoters of indicated genes. IgG is shown as a negative control.

(**I**) Glycolytic proton efflux rate (glycoPER) analyzed in *FTO* depleted KOPTK1 cells with or without the expression of *ELK3*.

(**J**) Glucose uptake (left) and lactate secretion (right) assessed in *FTO* depleted T-ALL cells with or without the expression of ELK3, normalized to the same total protein.

Data are presented as means (±SD) of technical triplicates, and the experiments were independently repeated at least twice with the similar results (A, D, G, H, I, J). Data were analyzed by one-way ANOVA with Tukey's multiple comparisons (A, J) and unpaired two-tailed Student's t-test (D, G, H). $^{**}P < 0.01$ and $^{***}P < 0.001$.

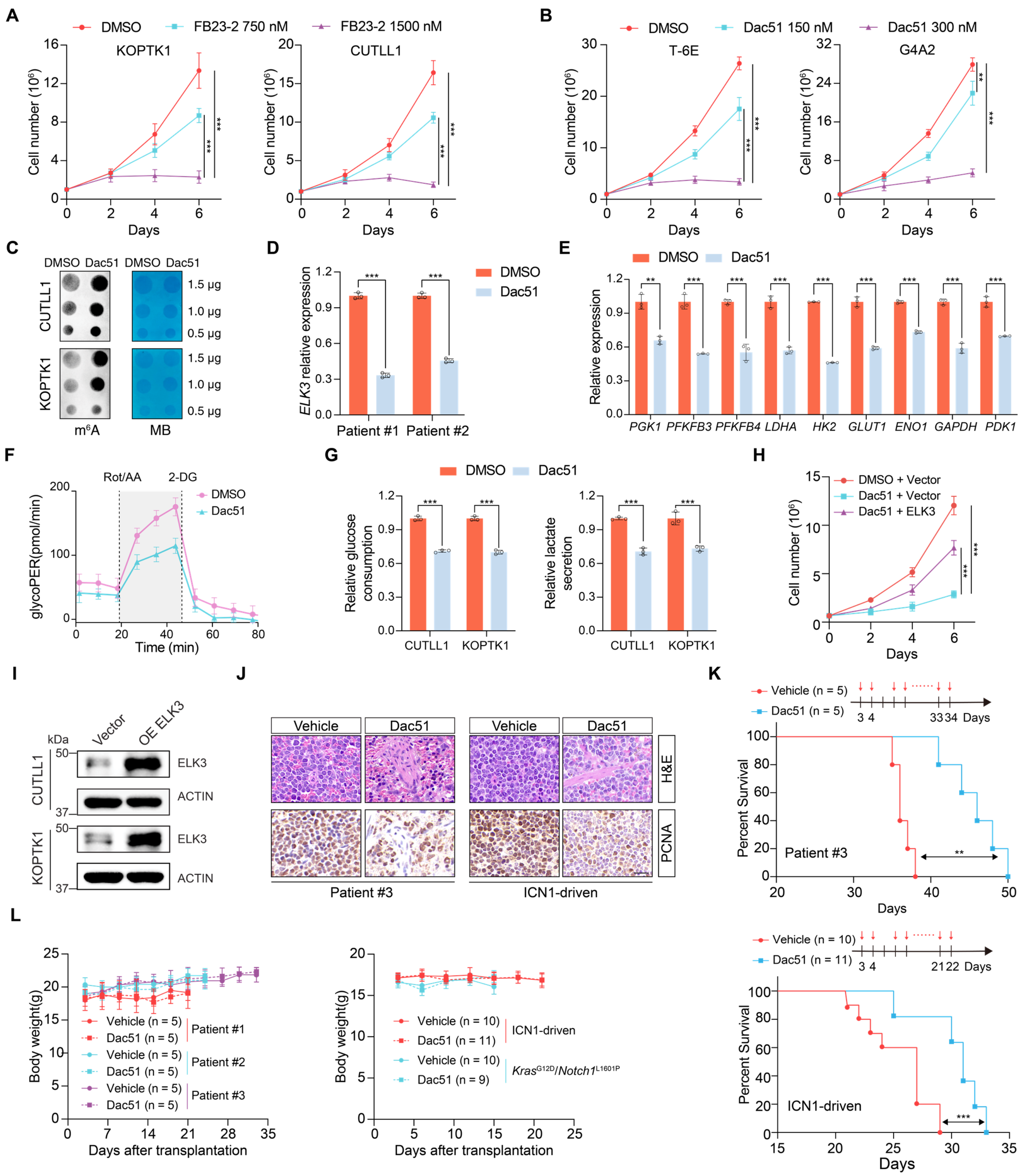
A
DMSO
FB23-2 750 nM
FB23-2 1500 nM
KOPTK1
CUTLL1
Cell number (10^6)
Days
B
DMSO
Dac51 150 nM
Dac51 300 nM
T-6E
G4A2
C
DMSO Dac51 DMSO Dac51
CUTLL1
KOPTK1
1.5 μg
1.0 μg
0.5 μg
m6A
MB
D
ELK3 relative expression
DMSO
Dac51
Patient #1
Patient #2
E
Relative expression
PGK1 PFKFB3 PFKFB4 LDHA HK2 GLUT1 ENO1 GAPDH PDK1
F
Rot/AA
2-DG
glycoPER(pmol/min)
Time (min)
G
Relative glucose consumption
Relative lactate secretion
CUTLL1
KOPTK1
H
DMSO + Vector
Dac51 + Vector
Dac51 + ELK3
I
kDa
Vector
OE ELK3
ELK3
ACTIN
J
Vehicle
Dac51
H&E
PCNA
Patient #3
ICN1-driven
K
Vehicle (n = 5)
Dac51 (n = 5)
Percent Survival
Vehicle (n = 10)
Dac51 (n = 11)
L
Body weight(g)
Days after transplantation
Patient #1
Patient #2
Patient #3
Vehicle (n = 10)
Dac51 (n = 11)
Dac51 (n = 9)
KrasG12D/Notch1L1601P

Fig. S8. FTO inhibitor Dac51 exhibits strong anti-leukemia efficacy *in vitro* and *in vivo*.

(**A**) Growth curves of KOPTK1 and CUTLL1 cells treated with varying doses of FB23-2 as shown.

(**B**) Growth curve of murine T-ALL cell lines treated with varying doses of Dac51.

(**C**) m^6A dot blot assay assessing the global m^6A abundance upon Dac51 (300 nM) treatment for 36 hours. Methylene blue (MB) staining was used as the loading control.

(**D**) qPCR analysis showing *ELK3* mRNA levels in two individual human primary T-ALL cells upon treatment with Dac51 (300 nM) for 36 hours.

(**E**) qPCR analysis of glycolysis-related gene expression in KOPTK1 cells with or without Dac51 treatment (300 nM, 36 hours).

(**F**) CUTLL1 cells were treated with 300 nM Dac51 for 36 hours, followed by analysis of glycolytic proton efflux rate (glycoPER).

(**G**) CUTLL1 and KOPTK1 cells were treated with 300 nM Dac51 for 24 hours, followed by assessment of glucose uptake (left) and lactate secretion (right). The value was normalized to the same total protein.

(**H**) Growth curves of 300 nM Dac51 treated KOPTK1 cells with or without ectopic expression of *ELK3*.

(**I**) Immunoblot showing the overexpression (OE) of ELK3 in leukemia cells.

(**J**) Hematoxylin and eosin (H&E) and immunohistochemistry staining of spleens from T-ALL PDX (Patient #3) or murine primary ICN1-driven murine T-ALL cells at 35 and 20 days post-transplantation, respectively. The scale bar represents 20 μm. PCNA: proliferating cell nuclear antigen.

(**K**) Kaplan-Meier survival curves of T-ALL mice in (J).

(**L**) Body weights of all T-ALL PDX and allografts after treatment with Dac51 as shown in Fig. 6G.

Data are presented as the mean (± SD) from two biological replicates, each performed with three technical replicates (A, B, H). Data are presented as the mean (± SD) of technical triplicates, and experiments were independently repeated at least twice with similar results (D-G). Data were analyzed by two-way ANOVA with Bonferroni's multiple comparisons (A, B, H) , unpaired two-tailed Student's t-test (D, E, G) and log-rank test (K). $^{**}P < 0.01$ and $^{***}P < 0.001$.

Table S1. Key features of three human primary T-ALL patients.

ID	T-ALL subtype	Mutational Status			
		NOTCH1	BCL11B	FBXW7	PTEN
Patient #1	*LMO2*	MUT (HD)	MUT	MUT	WT
Patient #2	*TAL 1*	MUT (HD&PEST)	WT	MUT	MUT
Patient #3	*TAL 1*	MUT (HD&PEST)	MUT	WT	MUT

Table S2. Primers and shRNAs used in this study.

Name	Sequence 5'-3'
**qPCR primers for human genes**	
FTO-qPCR-F	CCAGAACCTGAGGAGAGAATGG
FTO-qPCR-R	CGATGTCTGTGAGGTCAAACGG
ELK3-qPCR-F	ATCTGCTGGACCTCGAACGA
ELK3-qPCR-R	TTCTGCCCGATCACCTTCTTG
ACTB-qPCR-F	AGCCTCGCCTTTGCCGA
ACTB-qPCR-R	GCGCGGCGATATCATCATC
GAPDH-qPCR-F	GTCTCCTCTGACTTCAACAGCG
GAPDH-qPCR-R	ACCACCCTGTTGCTGTAGCCAA
PSIP1-qPCR-F	CGATTCCGAGCCGTTGAGA
PSIP1-qPCR-R	TCCCGACGCGCCTGC
TADA3-qPCR-F	CCTTGCAGTTCCACGACTTCA
TADA3-qPCR-R	TCAGGAATCGTCTGTCACCTTT
MAML3-qPCR-F	CACAGCGGAATCCATACCCAGT
MAML3-qPCR-R	ATGCCTGCGTTCTGTGCCATCA
HSF4-qPCR-F	GGACCAGTTTCCTCGTAAGCGA
HSF4-qPCR-R	CTCACCACCTTCCGAAAACCGT
RUNX3-qPCR-F	GGCAATGACGAGAACTACTCCG
RUNX3-qPCR-R	GATGGTCAGGGTGAAACTCTTCC
SP140-qPCR-F	GGAAAGTGACCAAGCATGTGGC
SP140-qPCR-R	TCTCGTTCCCATTCTGCTCCAG
BHLHE40-qPCR-F	TAAAGCGGAGCGAGGACAGCAA
BHLHE40-qPCR-R	GATGTTCGGGTAGGAGATCCTTC
ZNF460-qPCR-F	GACATTTACCCAGGAGGAGTGG
ZNF460-qPCR-R	GCCAGATGAGAAGGGATAGGCT
BCL6-qPCR-F	CATGCAGAGATGTGCCTCCACA
BCL6-qPCR-R	TCAGAGAAGCGGCAGTCACACT
KLF2-qPCR-F	CCAAGAGTTCGCATCTGAAGGC
KLF2-qPCR-R	CCGTGTGCTTTCGGTAGTGGC
HELZ2-qPCR-F	GAGGTGCATCTGTGTCGTTTCC
HELZ 2-qPCR-R	CAGGATCTCAAAACTGCCGACAG
PGK1-qPCR-F	CCGCTTTCATGTGGAGGAAGAAG

PGK1-qPCR-R	CTCTGTGAGCAGTGCCAAAAGC
PFKFB3-qPCR-F	GGCAGGAGAATGTGCTGGTCAT
PFKFB3-qPCR-R	CATAAGCGACAGGCGTCAGTTTC
PFKFB4-qPCR-F	ACAGTGATGAGGCTACGG
PFKFB4-qPCR-R	ATGCGGCTCTGGATGTG
GLUT1-qPCR-F	GGCCAAGAGTGTGCTAAAGAA
GLUT1-qPCR-R	ACAGCGTTGATGCCAGACAG
ENO1-qPCR-F	TGGTGTCTATCGAAGATCCCTT
ENO1-qPCR-R	CCTTGGCGATCCTCTTTGG
LDHA-qPCR-F	GGATCTCCAACATGGCAGCCTT
LDHA-qPCR-R	AGACGGCTTTCTCCCTCTTGCT
HK2-qPCR-F	GAGCCACCACTCACCCTACT
HK2-qPCR-R	CCAGGCATTCGGCAATGTG
PDK1-qPCR-F	CTGTGATACGGATCAGAAACCG
PDK1-qPCR-R	TCCACCAAACAATAAAGAGTGCT
**Primers for m^6A-qPCR**	
ELK3-m^6A-qPCR-F	CCTCACCCCAGCCTTCTTCAC
ELK3-m^6A-qPCR-F	GCACTGGCATGTGGCCATT
**Primers for ELK3 ChIP-qPCR**	
PGK1-ChIP-F	GGGAAGGTTCCTTGCGGTTC
PGK1-ChIP-R	GTCCGTCTGCGAGGGTACTA
LDHA-ChIP-F	GACGTCAGCATAGCTGTTCC
LDHA-ChIP-R	TACCGGGAATGCACGTCG
GAPDH-ChIP-F	CCCTCTCCAGCTCCTCAAGT
GAPDH-ChIP-R	CAAAGAGGGAGCTCAGTGCC
PFKFB3-ChIP-F	CCCTTCAGGAAAGGTAGGAGT
PFKFB3-ChIP-R	GAGCGCGTCTCGCTTGG
HK2-ChIP-F	GGGAGCGGAAAAAGTTTGGG
HK2-ChIP-R	CAGGGAAAAAGGGACTGGGG
ENO1-ChIP-F	AGCCTCTCAACGACTCGACG
ENO1-ChIP-R	CACTCAGCGTCGCTCTGG
GLUT1-ChIP-F	ACTTTGATCAGACCTTTGGACTGG
GLUT1-ChIP-R	ATCATCTGACTTCACCTTATTTCCT
PFKFB4-ChIP-F	CTATGCAAATCAGCGCGGG
PFKFB4-ChIP-R	ACTCCGGATTGTACTGGTCC
PDK1-ChIP-F	ACTTCTACGCGCGCTTCTC
PDK1-ChIP-R	AAAAAGGCCCAAGGTCCCG
**Primers for H3K27ac ChIP-qPCR**	
PGK1-ChIP-F	CAAACCTCTTTGGCCGGAGC
PGK1-ChIP-R	CGAAGGGTGACTTCGGGTG
LDHA-ChIP-F	AACTGCCTCTGGTTCTGCTG
LDHA-ChIP-R	CATGGTTCCCGCTTAGAGCA

GAPDH-ChIP-F	CACCCTGCCCTCAATATCCC
GAPDH-ChIP-R	AAGGCACTCCTGGAAACCTG
PFKFB3-ChIP-F	GAAAGGTAGGAGTCCCGGTG
PFKFB3-ChIP-R	CCGGGTGCACAGTGCTT
HK2-ChIP-F	GGGCTCTGGCAAAGTGGTCTG
HK2-ChIP-R	CACTCGCCGGCTGTGCTT
ENO1-ChIP-F	GCGGTAGGGTCCATTGCTTC
ENO1-ChIP-R	GAACCCCGAATTAGGGACACG
GLUT1-ChIP-F	GGGGATGGGCTTCGGATCTA
GLUT1-ChIP-R	AGGAGGCCATGTGCTCAGT
PFKFB4-ChIP-F	CCCCACGGGAATTGACACAG
PFKFB4-ChIP-R	CACCTCCTCCCAGAGGACC
PDK1-ChIP-F	AGATTGGGAGGGCAGAGGAA
PDK1-ChIP-R	ACTAAGAGGCGAGGCGGTAT
**Target sequences for shRNA**	
shControl	TTCGATCTCAATTGCTATCGA
shFTO#1	TCACGAATTGCCCGAACATTA
shFTO#2	TCACCAAGGAGACTGCTATTT
shELK3#1	ACTCGTCCTTCACCATTAATT
shELK3#2	TGTTGAGGCTGTGGGTATAAA
shYTHDF2	GATGGATTAAACGATGATGAT
**Primers for genotyping**	
Alkbh5-F	GAGTGACAATGGAAATCACCAGGGT
Alkbh5-R	GGATGAAGCCTCATCAGGAGAACAGT
Fto-F	GATCAAAGGCCAGTTATTCTG
Fto-R	GGGTTCTTACCTTGCCAGTC
Lck-Cre-F	TGTGAACTTGGTGCTTGAGG
Lck-Cre-R	CAGGTTCTTGCGAACCTCAT
Mx1-Cre-P1	CGGTTATTCAACTTGCACCA
Mx1-Cre-P2	GACAAAATGGTGAAGGTCGG
Mx1-Cre-P3	CAAAGGCGGAGTTACCAGAG
Mx1-Cre-P4	GTGAGTTTCGTTTCTGAGCTCC
**qPCR primers for mouse genes**	
Alkbh5-qPCR-F	CGCGGTCATCAACGACTACC
Alkbh5-qPCR-R	ATGGGCTTGAACTGGAACTTG
Fto-qPCR-F	GCCTCGGTTTAGTTCCACTCAC
Fto-qPCR-R	GTCGCCATCGTCTGAGTCATTG
Elk3-qPCR-F	ACACTTCTGGAGCAGCCTTAGTC
Elk3-qPCR-R	CATGTGACCGTTGAGCAGTGTG
Actb-qPCR-F	AGTGTGACGTTGACATCCGT
Actb-qPCR-R	GCAGCTCAGTAACAGTCCGC
